# Supplementary material for: Machine learning-based prediction reveals kinase MAP4K4 regulates neutrophil differentiation through phosphorylating apoptosis-related proteins
Source: PLoS Comput Biol. 2025 Mar 17;21(3):e1012877. doi: 10.1371/journal.pcbi.1012877 (PMC11957395; doi:10.1371/journal.pcbi.1012877)
Supplement: S1 Text — (DOCX) [file pcbi.1012877.s001.docx]

**S1 Text: Description of *in silico* knockout process by OntoVAE**

**Architecture of OntoVAE**

OntoVAE[1] is a modified VAE whose latent space and decoder are designed to incorporate any biological ontology, such as Gene Ontology (**Figure A in S1 Text**). In this model, each node in the latent space and decoder represents an ontology term, with root terms in the latent space and more specific terms progressing through the decoder. To avoid a meaningless 1D latent space, it trims the ontology by removing the root and highly general terms (those with >1000 annotated genes). It also removes overly specific terms (those with <30 annotated genes). The decoder of OntoVAE is linear, prioritizing interpretability over reconstruction accuracy. It is sparse, as connections are only modeled between parent and children ontology terms as well as between terms and annotated genes. To preserve pathway activity directionality, OntoVAE restricts decoder weights to be positive. The model uses a layer concatenation process (akin to DenseNet[2] skip connections) to account for relationships across non-adjacent layers and between any layer and the reconstruction layer (**Figure B in S1 Text**). Each ontology term is represented by three neurons to model more complex relationships.

The loss function is the same as VAE[3], consisting of two components: the reconstruction error and the Kullback-Leibler (KL) divergence. The formula is as follows:

$$L\left( \theta,\phi;x \right)=E_{q_{\phi}\left( z | x \right)}[{logp}_{\theta}\left( x | z \right)\boldsymbol{]}-D_{KL}[q_{\phi}(z|x)||p\left( z \right)\boldsymbol{]}$$

The first term is the reconstruction error, representing the log-likelihood of reconstructing the input data given the latent variable $z$, and the second term is the KL divergence, which measures the difference between the variational posterior $q_{\phi}\left( z | x \right)$and the prior $p\left( z \right)$.

**The datasets used for training OntoVAE**

To identify genes that affect neutrophil differentiation, we specifically used 2040 bulk RNA-seq datasets of immune cells from ImmuNexUT[4] and Atlas Human Lymphocytes[5]. Then we performed batch correction to obtain a gene expression matrix as the input of the OntoVAE. The datasets were also summarized in **S1 Table**.

**Training details**

We trained OntoVAE with a batch size of 128, a dropout of 0.2 in the hidden layer of the encoder, a dropout of 0.5 on the latent space layer, and a weighting coefficient of 1x10^-4^ on the KL loss. We performed model training on 80% of the samples, while the remaining 20% were used for validation. The model was trained for 300 epochs, with a stable validation loss (**S1H Fig**). We used AdamW as the optimizer with a learning rate of 1x10^-4^. The exact layer compositions of the model are given in **Table A in S1 Text**.

**Accuracy of *in silico* knockouts**

After training, OntoVAE can capture the relationships between gene expression and term activity. So the activations of the neurons in the latent space and decoder can be directly interpreted as term activities. We then retrieved the activities of all terms in the latent space and decoder for each sample and looked at some example terms to see if they were more active in the expected cells. For instance, the ‘neutrophil activation’ pathway is especially active in neutrophils, and the ‘regulation of mononuclear cell proliferation’ pathway is active in monocytes (**S1H Fig**).

Next, we used OntoVAE for *in silico* gene knockouts (**Figure C in S1 Text**). To simulate a gene knockout, the input value for a given gene can be set to zero before passing the samples through the trained model to obtain the activations at each ontology term. Afterward, paired Wilcoxon tests can be conducted for each term in both the latent space and the decoder (comparing pre-knockout versus post-knockout conditions) to identify the terms that are significantly affected by the knockout. To test the validity, we performed *in silico* knockouts of the *ELANE*[6] and *SYK*[7], those two genes have been reported to play important roles in neutrophils and B cells, respectively. The results showed that neutrophil-related and B cell-related terms were affected significantly and fitted the reported studies[8–11] (**S2A Fig**), suggesting that using the data in **S1** **Table**, together with the OntoVAE, can accurately mimic the *in silico* knockouts.


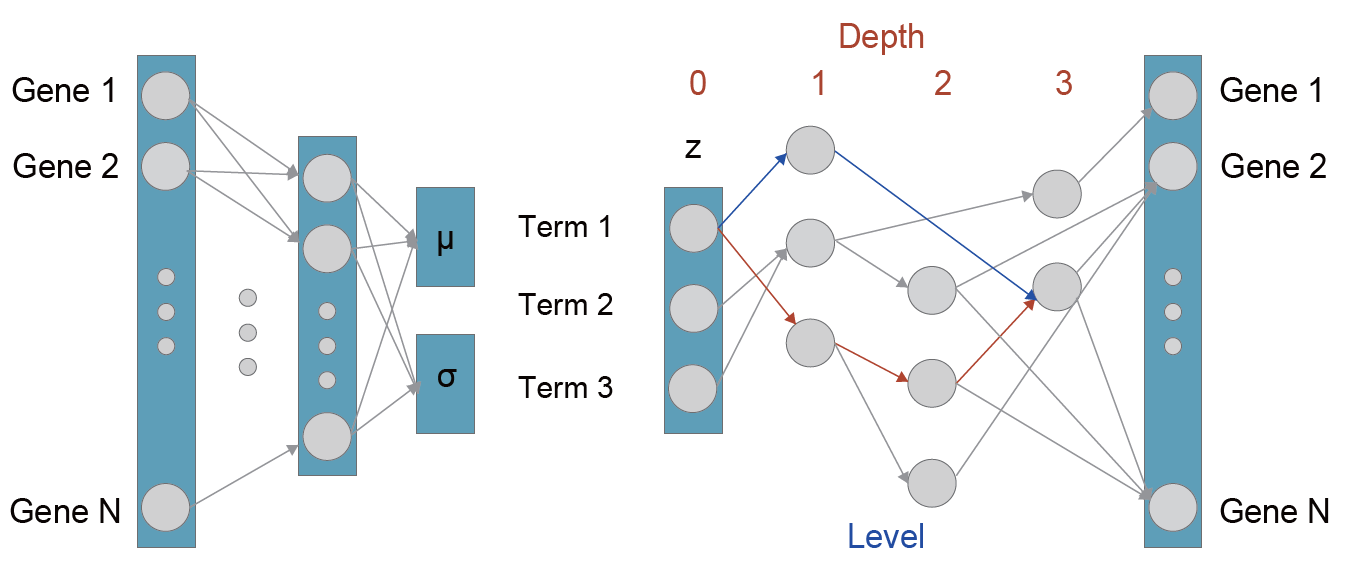


**Figure A**. A schematic of OntoVAE is shown, where a non-linear encoder is connected to a masked, multi-layer linear decoder that reflects a biological ontology. The latent space includes the root terms of the ontology, while each layer in the decoder corresponds to a specific depth of the ontology. Here, 'depth' refers to the longest possible path from a node to a root node, and 'level' refers to the shortest possible path from a node to a root node.


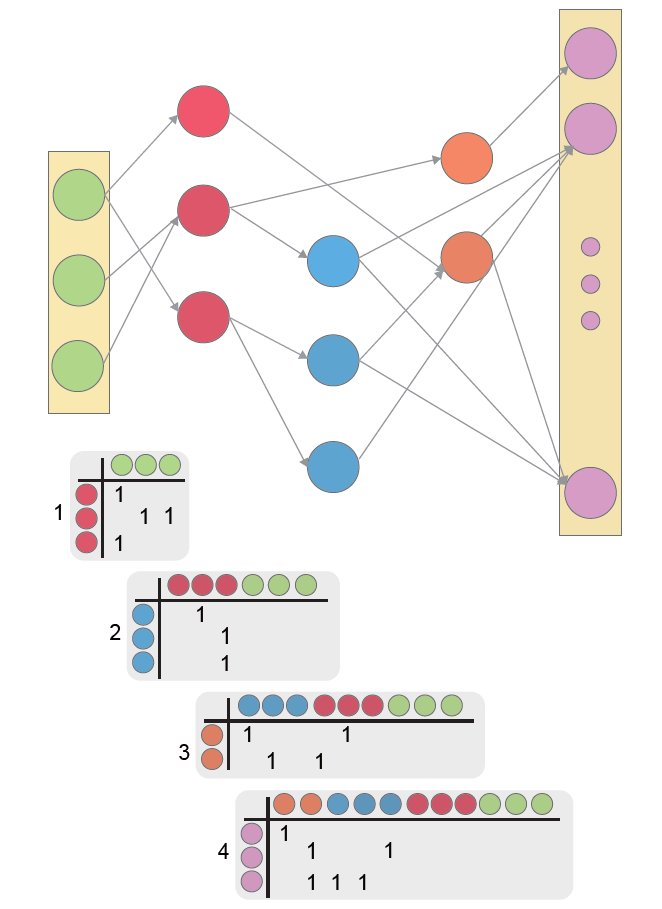


**Figure B.** Schematic drawing of skip connection modeling through concatenations. Binary masks represent the connections between the latent space layer (green), decoder layers (red, blue, orange), and the reconstruction layer (pink). At each step, the output from the previous layer is concatenated with the current layer. Mask 1 captures the connections between the green (latent space) and red layers, and these two layers are concatenated, enabling Mask 2 to model the connections from both the green and red layers to the blue layer. Mask 3 models the connections between the orange layer and all preceding layers, while Mask 4 models the connections between the pink (reconstruction) layer and all previous layers.

**Table A.** Structure of OntoVAE model that was trained on 2040 immune cell samples with trimming thresholds 1000 and 30. n = number of neurons per term, we used n = 3 in the study. The latent space is represented by two separate layers: mu and logvar. The Depth column indicates the depth level of the ontology corresponding to each network layer, while the Layer column outlines the structure of each layer. The Input dim and Output dim columns specify the number of features that each layer uses as input and output, respectively. These dimensions are determined by the trimmed ontology, the number of genes annotated to it, and the number of neurons allocated per ontology term (n). Since the decoder layers are concatenated at each step, the Input dim for the current layer is always the sum of the previous layer’s output and the current layer’s input. The Output dim column indicates the number of GO terms at each depth level, for example, 585 terms at layer 0, 14 terms at layer 1, and so on. The Connections column specifies the number of connections present in the binary mask linking the layers, for instance, 15 connections between layer 0 and layer 1, 76 connections between the concatenation of layers 0 and 1 to layer 2, and so forth. The total number of connections depends on the value of n.


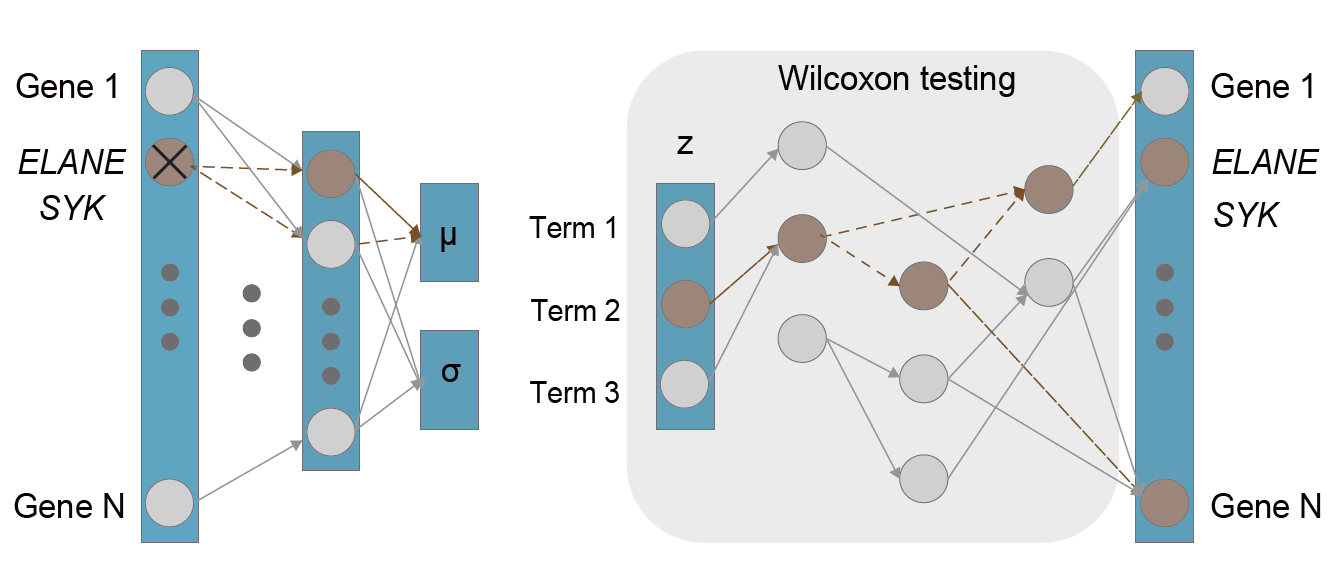


**Figure C**. OntoVAE can predict pathway alternations of a gene knockout (here: *ELANE* or *SYK*). In our study, *in silico* knockouts of those two genes have been performed in neutrophils or B cells, respectively. Schematic drawing of how the model can be used for *in silico* investigation of gene knockouts. The input value for a gene (here: *ELANE* or *SYK*) can be set to zero before running samples through the trained model and obtaining their activations at each term. Paired Wilcoxon tests can be performed for all the terms in latent space and decoder (pre-knockout versus post-knockout) to identify the affected term.

**References:**

1. Doncevic D, Herrmann C. Biologically informed variational autoencoders allow predictive modeling of genetic and drug-induced perturbations. Bioinformatics. 2023;39. doi:10.1093/bioinformatics/btad387

2. Huang G, Liu Z, Maaten L van der, Weinberger KQ. Densely Connected Convolutional Networks. arXiv; 2018. doi:10.48550/arXiv.1608.06993

3. Kingma DP, Welling M. Auto-Encoding Variational Bayes. arXiv; 2022. doi:10.48550/arXiv.1312.6114

4. Ota M, Nagafuchi Y, Hatano H, Ishigaki K, Terao C, Takeshima Y, et al. Dynamic landscape of immune cell-specific gene regulation in immune-mediated diseases. Cell. 2021;184: 3006-3021.e17. doi:10.1016/j.cell.2021.03.056

5. Bonnal RJP, Ranzani V, Arrigoni A, Curti S, Panzeri I, Gruarin P, et al. De novo transcriptome profiling of highly purified human lymphocytes primary cells. Sci data. 2015;2: 150051. doi:10.1038/sdata.2015.51

6. Rao S, Yao Y, Soares de Brito J, Yao Q, Shen AH, Watkinson RE, et al. Dissecting <em>ELANE</em> neutropenia pathogenicity by human HSC gene editing. Cell Stem Cell. 2021;28: 833-845.e5. doi:10.1016/j.stem.2020.12.015

7. Zhang S, Wang L, Lu Y, Guo C, Zhang T, Zhang L. Targeting spleen tyrosine kinase (SYK): structure, mechanisms and drug discovery. Drug Discov Today. 2024;30: 104257. doi:10.1016/j.drudis.2024.104257

8. Weinrauch Y, Drujan D, Shapiro SD, Weiss J, Zychlinsky A. Neutrophil elastase targets virulence factors of enterobacteria. Nature. 2002;417: 91–94. doi:10.1038/417091a

9. Nanua S, Murakami M, Xia J, Grenda DS, Woloszynek J, Strand M, et al. Activation of the unfolded protein response is associated with impaired granulopoiesis in transgenic mice expressing mutant Elane. Blood. 2011;117: 3539–3547. doi:10.1182/blood-2010-10-311704

10. Ackermann JA, Nys J, Schweighoffer E, McCleary S, Smithers N, Tybulewicz VLJ. Syk tyrosine kinase is critical for B cell antibody responses and memory B cell survival. J Immunol. 2015;194: 4650–4656. doi:10.4049/jimmunol.1500461

11. Chen L, Monti S, Juszczynski P, Daley J, Chen W, Witzig TE, et al. SYK-dependent tonic B-cell receptor signaling is a rational treatment target in diffuse large B-cell lymphoma. Blood. 2008;111: 2230–2237. doi:10.1182/blood-2007-07-100115
