# Supplementary figures and images for: Machine learning-based prediction reveals kinase MAP4K4 regulates neutrophil differentiation through phosphorylating apoptosis-related proteins
Source: PLoS Comput Biol. 2025 Mar 17;21(3):e1012877. doi: 10.1371/journal.pcbi.1012877 (PMC11957395; doi:10.1371/journal.pcbi.1012877)

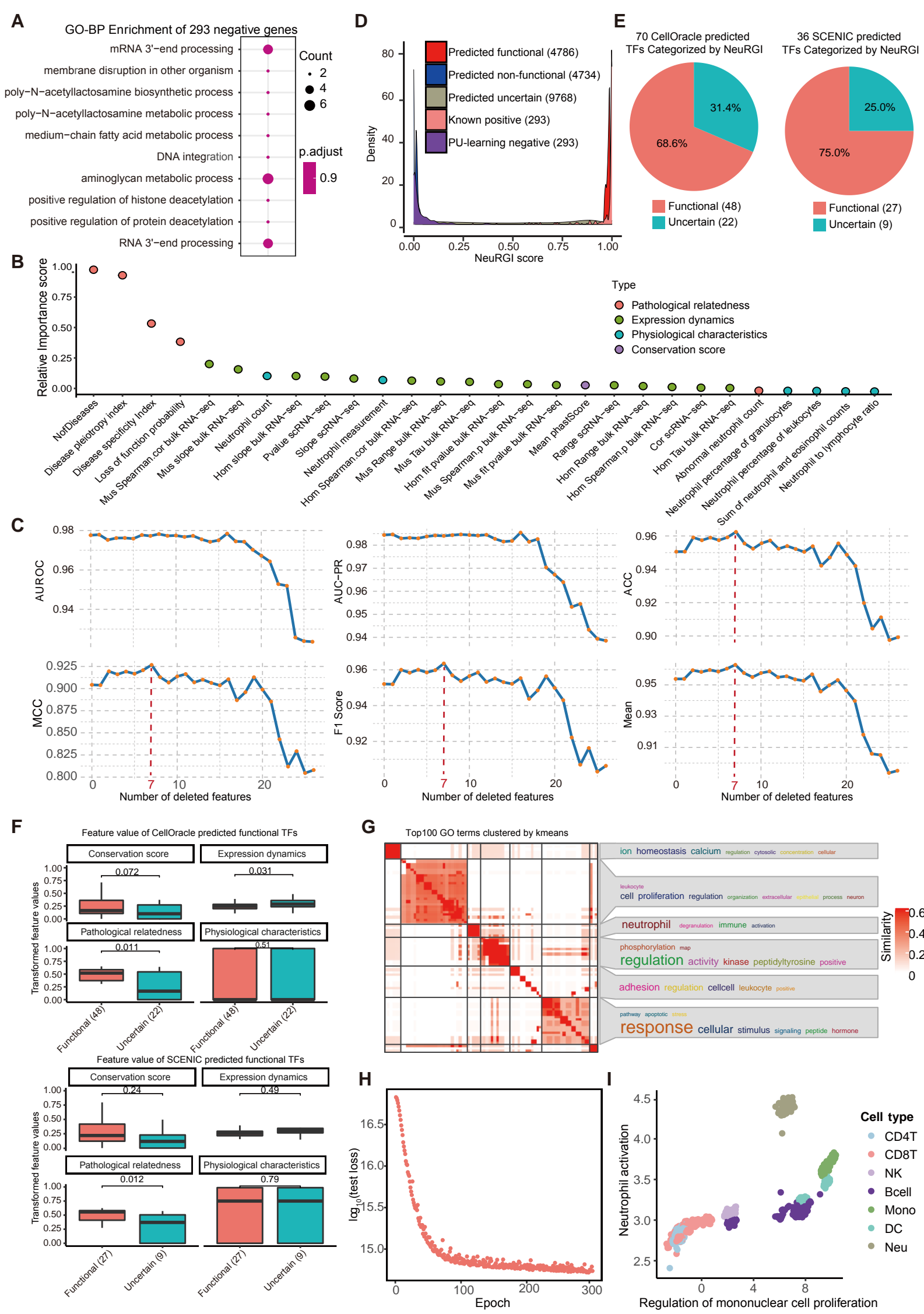

Supplement: S1 Fig — (A) Gene Ontology Biological Processes (GO-BP) enrichment of 293 PU-learning negative genes. These genes are not related to any neutrophil pathway. (B) Dot plot showing the feature importance of the baseline model using Gini coefficient with all features included. (C) Feature ablation studies. The line plots depict changes in the model’s evaluation metrics—AUC, AUC-PR, ACC, MCC, F1 Score, and Mean (average of these five metrics)—as features are sequentially removed in reverse order of their importance. The results indicate that the model achieves optimal performance when the last seven features are excluded. (D) Density distribution of NeuRGI scores across different gene categories, including predicted function (red, 4,786 genes), predicted non-function (blue, 4,734 genes), predicted uncertain (grey, 9,768 genes), known positive (light red, 293 genes), and PU-learning negative (purple, 293 genes). (E) Pie chart illustrating the NeuRGI classification of 70 CellOracle-predicted TFs and 36 SCENIC-predicted TFs. (F) The boxplot illustrates the transformed feature values for 70 CellOracle-predicted TFs (up) and 36 SCENIC-predicted TFs (down) in four feature groups. The p value was calculated using the Student’s t-test. (G) Heatmap illustrates the results of Kmeans clustering applied to the top 100 GO-BP terms associated with a set of 4,786 predictive functional genes. Rows and columns represent unique GO terms, with color intensity indicating similarity, where darker red denotes greater similarity. (H) The trend of test loss in OntoVAE model training. After 300 epochs of model training, the loss value no longer decreases and remains stable. (I) Scatter plot showing example pathway activity scores retrieved from OntoVAE model. The ‘neutrophil activation’ pathway is especially active in neutrophils and the ‘regulation of mononuclear cell proliferation’ pathway is especially active in monocytes. (PDF) [file pcbi.1012877.s017.pdf]

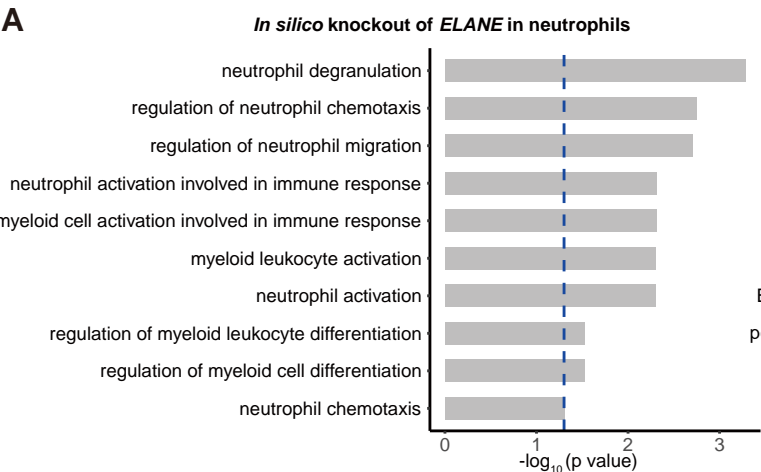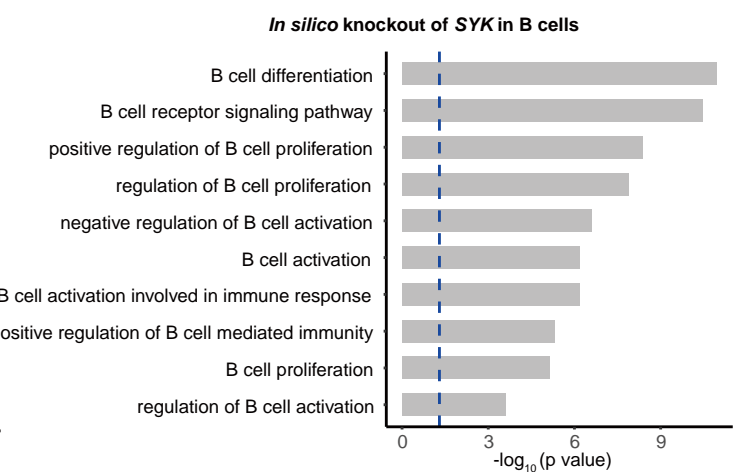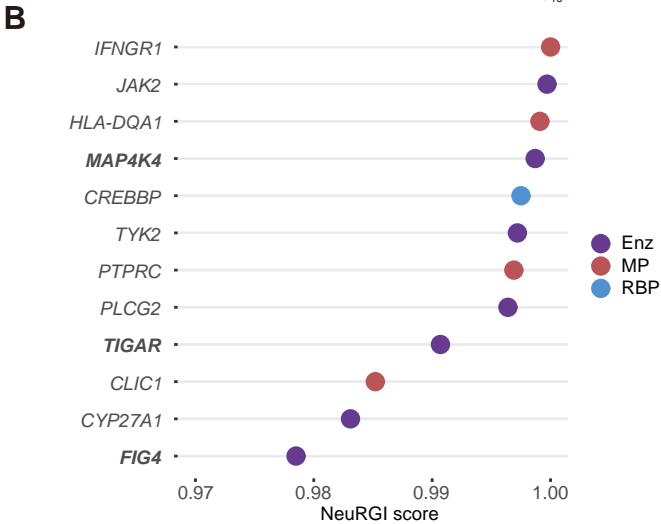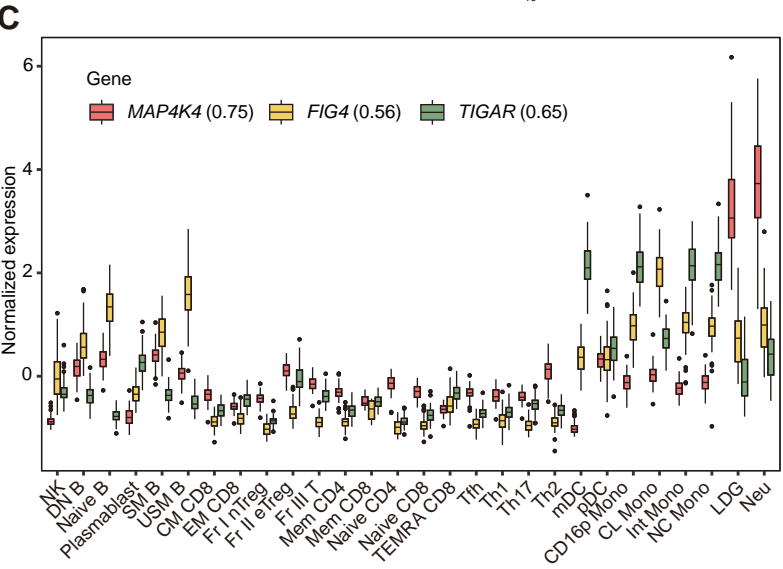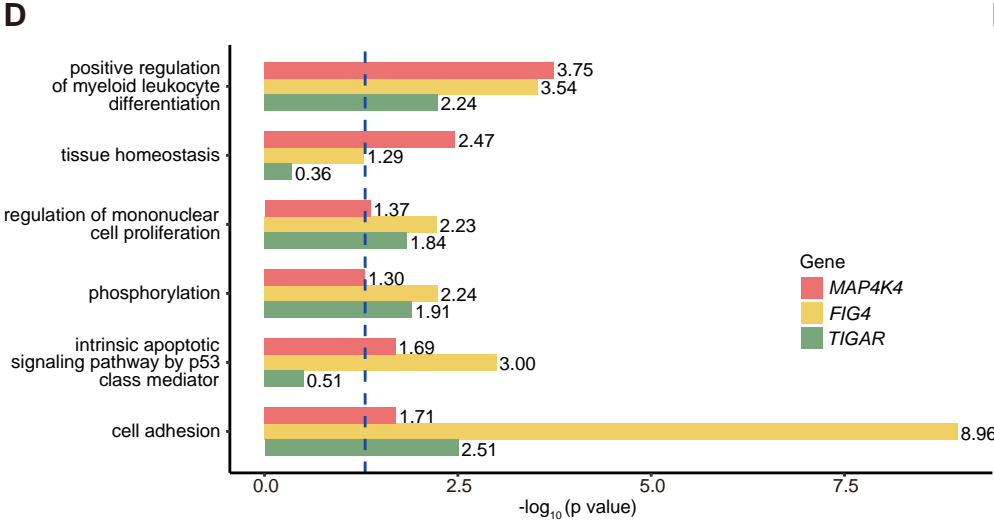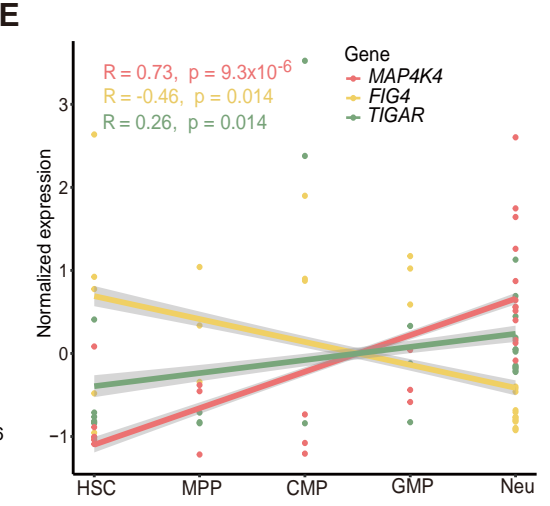

Supplement: S2 Fig — (A) In silico knockout of ELANE and SYK in neutrophils and B cells, respectively. Bar plots display the affected neutrophil and B cell related terms ranked by significance. The dotted line represents the significance threshold. (B) The dot plot showed the NeuRGI score of the top 12 genes. Genes displayed in bold indicate those with limited reports on their involvement in neutrophil differentiation or function. (C) Expression of MAP4K4, FIG4, and TIGAR in different immune cells from ImmuNexUT. The number behind genes represents cell specificity Tau value. (D) Bar plot shows main gene pathways affected by OntoVAE in silico KO of MAP4K4, FIG4, and TIGAR in neutrophils. In the ‘positive regulation of myeloid leukocyte differentiation’ pathway, MAP4K4 exhibits the lowest p value among the three genes. The dotted line represents the significance threshold. (E) Expression of MAP4K4, FIG4, and TIGAR in neutrophil differentiation. We set ‘time cut’ for cells at different differentiation stages, with HSC set as 1 and Neu as 5, and performed linear regression fitting for the expression of these 3 genes. R represents the Pearson correlation coefficient, and the p value was calculated by t-test. (PDF) [file pcbi.1012877.s018.pdf]

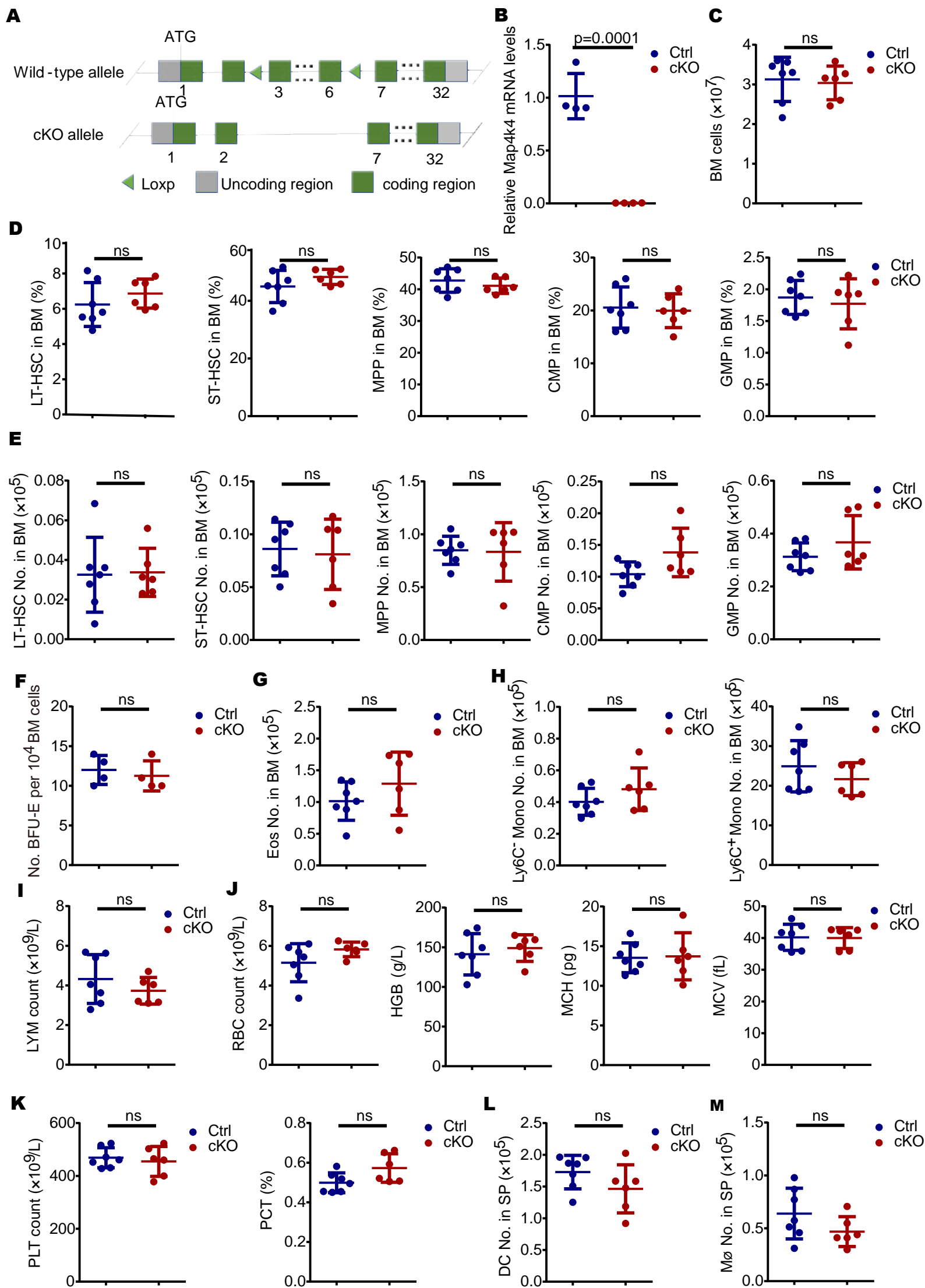

Supplement: S3 Fig — (A) Map4k4-deficient mice model. (B) RT-qPCR analysis of Map4k4 knockdown efficiency in mice BM cells (n=4; mean ± SD). Unpaired Student’s t-test. (C) Bone marrow cell numbers control (Ctrl) or Map4k4-cKO (cKO) mice; BM, bone marrow; (Ctrl n=7, cKO n=6; mean ± SD). (D) Percentage of hematopoietic stem and progenitor cells in Bone marrow of control (Ctrl) or Map4k4-cKO (cKO) mice; LT-HSC, Long-term hematopoietic stem cells; ST-HSC, Short-term hematopoietic stem cells; MPP, Multipotent blood progenitors; CMP, Common Myeloid Progenitor; GMP, Granulocyte-Macrophage Progenitor; BM, bone marrow; (Ctrl n=7, cKO n=6; mean ± SD). (E) Numbers of hematopoietic stem and progenitor cells in Bone marrow of control (Ctrl) or Map4k4-cKO (cKO) mice; LT-HSC, Long-term hematopoietic stem cells; ST-HSC, Short-term hematopoietic stem cells; MPP, Multipotent blood progenitors; CMP, Common Myeloid Progenitor; GMP, Granulocyte-Macrophage Progenitor; BM, bone marrow; (Ctrl n=7, cKO n=6; mean ± SD). (F) The number of primitive erythroid progenitor cells (BFU-E) colonies formed by 25,000 whole bone marrow cells from control (Ctrl) or Map4k4-cKO (cKO) BM, bone marrow; (n=4; mean ± SD). (G) Numbers of Eos cells in the Bone marrow of control (Ctrl) or Map4k4-cKO (cKO) mice; BM, bone marrow; Eso, eosinophils; (Ctrl n=7, cKO n=6; mean ± SD). (H) Numbers of Mon cells in the Bone marrow of control (Ctrl) or Map4k4-cKO (cKO) mice; BM, bone marrow; Mon, monocyte (Ctrl n=7, cKO n=6; mean ± SD). (I) PB LYM of control (Ctrl) or Map4k4-cKO (cKO) mice; PB, peripheral blood; LYM, lymphocyte (Ctrl n=7, cKO n=6; mean ± SD). (J) PB RBC numbers, HGB, MCH, and MCV of control (Ctrl) or Map4k4-cKO (cKO) mice; PB, peripheral blood; RBC, Red blood cell; HGB, Hemoglobin; MCH, Mean corpuscular hemoglobin; MCV, Mean corpuscular volume; (Ctrl n=7, cKO n=6; mean ± SD). (K) PB PLT, PCT of control (Ctrl) or Map4k4-cKO (cKO) mice; PB, peripheral blood; MPLT, Platelets; PCT, Plateletcrit; (Ctrl n=7, cKO n=6; mean ± SD). (L) [file pcbi.1012877.s019.pdf]

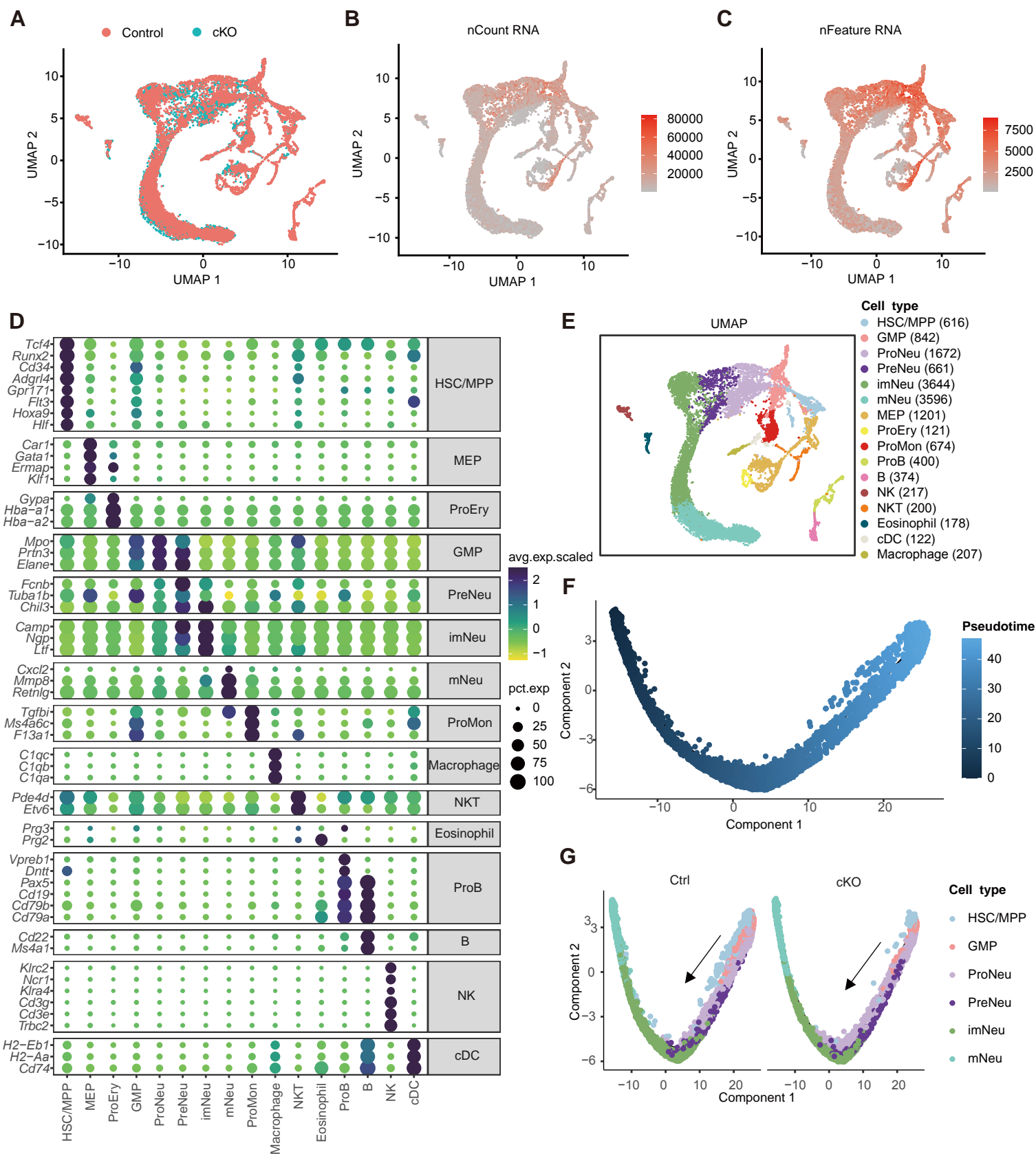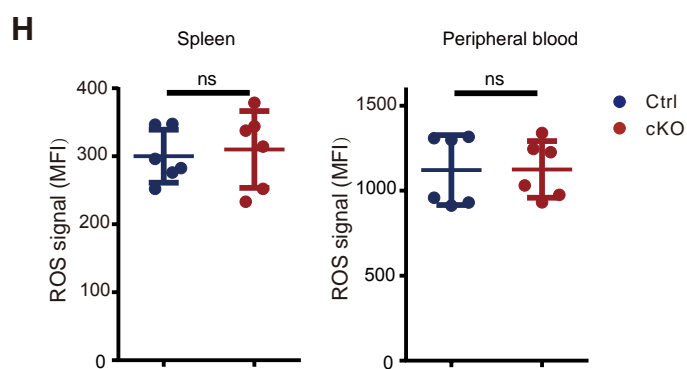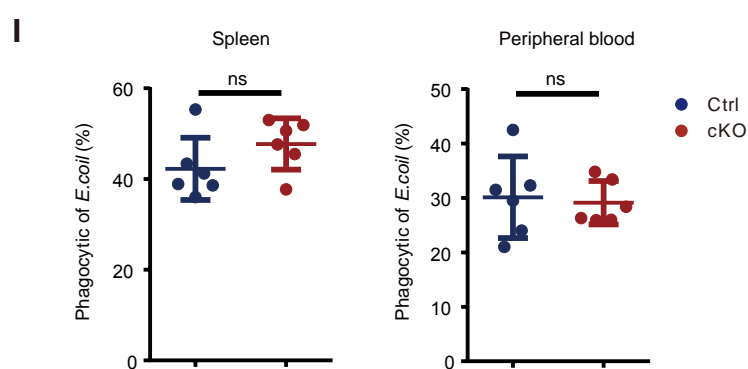

Supplement: S4 Fig — (A-C) UMAP of the 14,725 cells profiled here, with each cell color-coded for (A) Control or cKO, (B) the number of counts per barcode, and (C) the number of detected genes per cell. (D) Dot plot of marker genes. The dot plot shows the average expression level (the intensity of green) and percentage of expressed cells (the dot size). (E) UMAP of the 14,725 cells colored by cell type. (F) Monocle trajectories of neutrophils colored by pseudotime. (G) Monocle trajectories of neutrophils colored by cell type. Each dot represents a single cell. Cell orders are inferred from the expression of the most variable genes across all cells. Trajectory directions were determined by biological prior. (H) ROS generation of neutrophils in the spleen (Left) and peripheral blood (Right) of control (Ctrl) or Map4k4-cKO (cKO) mice; MFI, mean fluorescent intensity; ROS, reactive oxygen species; (n=6; mean ± SD). Mann-Whitney U test. (I) The percentage of phagocytic neutrophils in the spleen (Left) and peripheral blood (Right) of control (Ctrl) or Map4k4-cKO (cKO) mice; (n=6; mean ± SD). Mann-Whitney U test. (PDF) [file pcbi.1012877.s020.pdf]

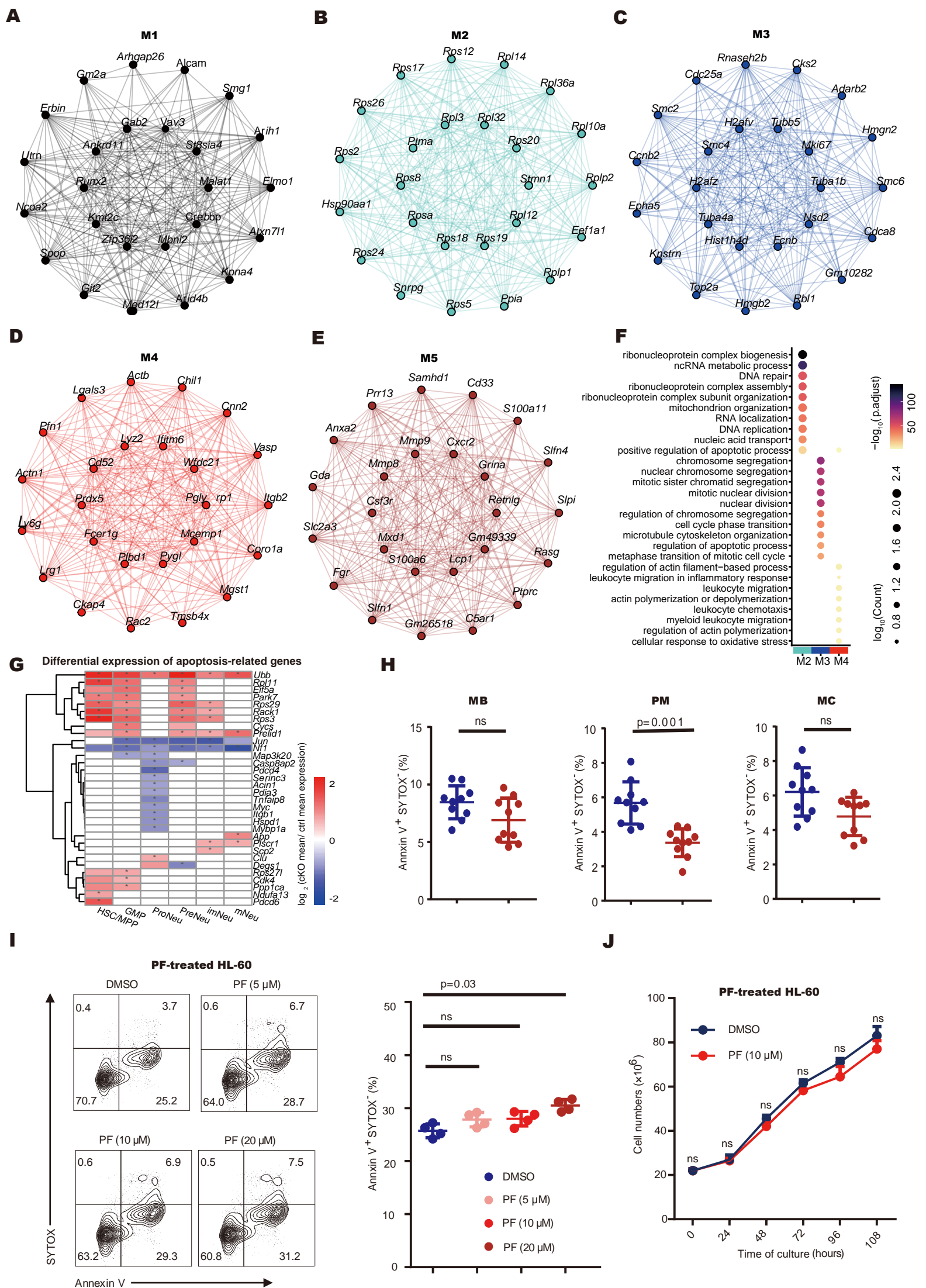

Supplement: S5 Fig — The top 25 hub genes ranked by kME are visualized. Nodes represent genes, and edges represent co-expression links. (F) Gene ontology enrichment analyses of hub genes from co-expression module M2 M3 M4. The dot size represented the number of genes. The color scale represented the adjusted p value. (G) Heatmap showing log2 (foldchange) in gene expression of the representative top300 apoptosis-related DEGs between control and cKO. The asterisks mean padjust < 0.05 in corresponding cells. (H) Percentages of annexin V+ and SYTOX- cells in bone marrow neutrophil progenitor cells of control (Ctrl) or Map4k4-cKO (cKO) mice; MB, myeloblasts, MB; PM, promyelocytes, PM; MC, myelocytes; BM, bone marrow (n=10; mean ± SD). Mann-Whitney U test. (I) Representative FACS analysis of HL-60 cells treated with 0-20 µM MAP4K4 inhibitor (PF-06260933, PF) for staining of annexin V and SYTOX was shown (left). percentages of annexin V+ and SYTOX- cells (right) (n=4; mean ± SD). Mann-Whitney U test. (J) Quantification of HL-60 cell numbers following treatment with a 10 µM concentration of the MAP4K4 inhibitor (PF-06260933, PF) was conducted over a period extending from 0 to 108 hours (n=4; mean ± SD). Mann-Whitney U test. (PDF) [file pcbi.1012877.s021.pdf]

**A**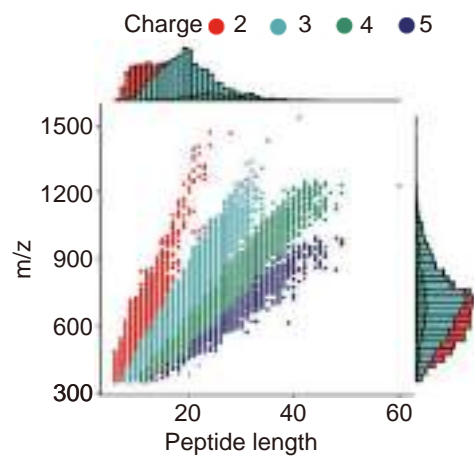**B**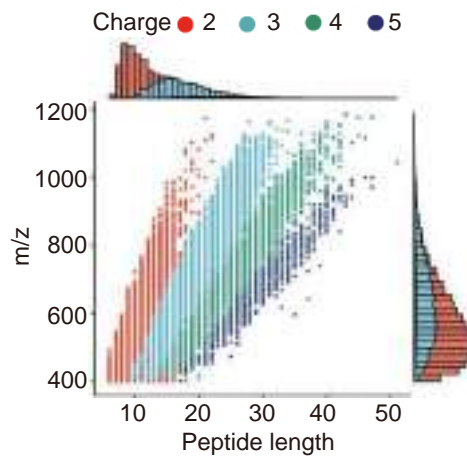**C**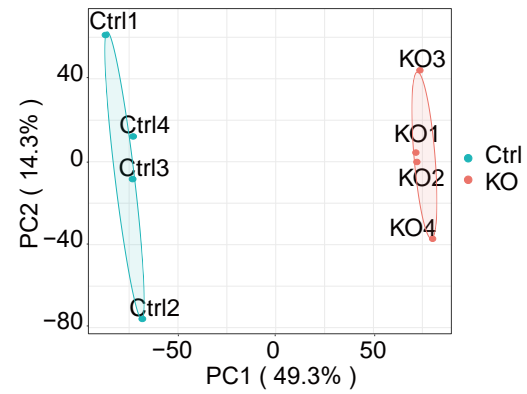**D**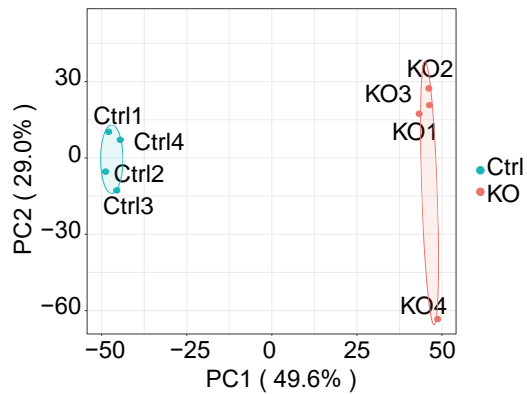**E**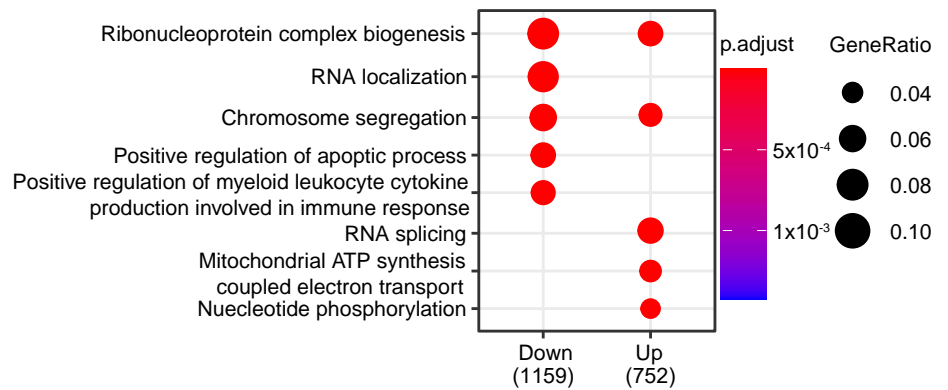**F**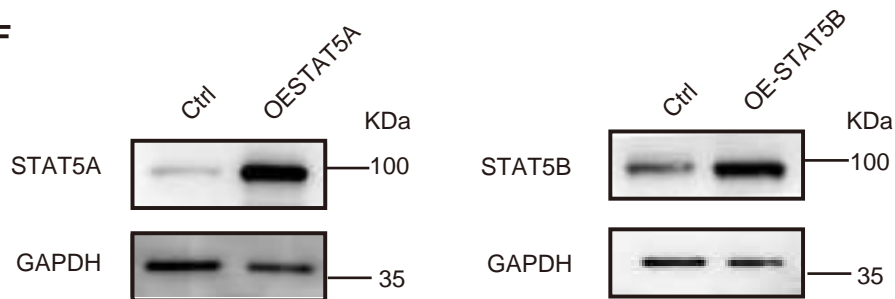

Supplement: S6 Fig — (A-B) The figure shows the distribution of peptide length and the corresponding m/z (mass-to-charge ratio) for peptides with different charge states, (A) for protein, and (B) for phosphorylation. Each point represents a peptide, with the color indicating its charge state. The histograms above and to the right of the scatter plot represent the distributions of peptide length and m/z, respectively, for peptides with different charge states. (C-D) The principal components analysis (PCA) results of 4 Ctrl samples (blue) and 4 MAP4K4 KO samples (red) for protein (C) and phosphorylation (D) from mass spectrometry. (E) GO-BP analysis of differential phosphorylation level proteins between Ctrl and MAP4K4 KO samples. Selected GO terms with Benjamini-Hochberg-corrected p values < 0.05 (one-sided Fisher’s exact test) are shown. The dot size represented the number of genes. The color scale represented the adjusted p value. (F) Western blot (WB) for STAT5A and GAPDH in Ctrl and STAT5A overexpression cells (left), for STAT5B and GAPDH in Ctrl and STAT5B overexpression cells (right). (PDF) [file pcbi.1012877.s022.pdf]
