# Supplementary material for: Machine learning-based prediction reveals kinase MAP4K4 regulates neutrophil differentiation through phosphorylating apoptosis-related proteins
Source: PLoS Comput Biol. 2025 Mar 17;21(3):e1012877. doi: 10.1371/journal.pcbi.1012877 (PMC11957395; doi:10.1371/journal.pcbi.1012877)
Supplement: Attachment — Submitted filename: Response to Reviewers.docx [file pcbi.1012877.s024.docx]

Lu Chen, Ph.D. Professor

Sichuan University, Chengdu, China 610041

Email: [luchen@scu.edu.cn](http://luchen@scu.edu.cn) Tel: (+86)18180609879

Jan. 16, 2025

**MS Submission ID:** PCOMPBIOL-D-24-01958
**MS title:** Machine learning-based prediction reveals kinase MAP4K4 regulates neutrophil differentiation through phosphorylating apoptosis-related proteins

Dear Dr. Murphy,

We would like to extend our heartfelt thanks to your invaluable assistance and express our gratitude to the reviewers for their positive comments and constructive suggestions. To address the concerns of you and the reviewers, we have conducted a methods comparison with CellOracle and SCENIC, performed feature ablation studies, updated the model to a neutrophil-specific version called NeuRGI, and carried out additional experiments. All these new data were incorporated into the manuscript, figures, supplementary tables, and supplementary note.

We have uploaded a point-by-point response to the reviewers' comments, along with the manuscript (both clean and track-change versions). Additionally, we have formatted the manuscript according to the *PLOS Computational Biology* article style, as recommended.

Thank you again for your kind consideration, detailed instructions, and continued support throughout the review process. We hope that the revision will satisfactorily address the comments and concerns of you and the reviewers.

Yours Sincerely,

Lu Chen (on behalf of all co-authors)

Professor, Sichuan University

**Point-by-point response**

**Editor**

**Both reviewers appreciate your study’s combination of computational modeling and collection of experimental support for computational predictions. However, both raise a range of issues ranging from relatively minor to quite significant. I believe that the most important issue to address is comparison with existing gene regulatory predictive models (issue 8 of Reviewer 1). I encourage you to respond to each reviewer concern either by making appropriate additions to the manuscript, or by providing careful explanations of the reasons that you have chosen not to.**

*Response:* Thanks for the positive comments on our work. We respond to each point raised by the reviewers, including the comparison with existing gene regulatory predictive models (issue 8 of Reviewer 1).

**Reviewer #1: This study presents a machine learning pipeline, MRGI (Myeloid Regulatory Gene Identifier), based on random forests to predict key genes involved in myeloid cell differentiation. The authors integrated features such as gene expression dynamics, physiological characteristics, pathological relevance, and conservation to train the model. The study highlights the MAP4K4 gene as a regulator of neutrophil differentiation and validates its function through in vivo and in vitro experiments, demonstrating that MAP4K4 modulates apoptosis-related protein phosphorylation during differentiation. However, I have several concerns, which are described in detail below.**

*Response*: Thank you for your thoughtful review and constructive suggestions. According to the suggestion of reviewer 2, we refined the model to be neutrophil-specific and consequently renamed our method MRGI (Myeloid Regulatory Gene Identifier) as NeuRGI (Neutrophil Regulatory Gene Identifier).

**1. In section "Step 1: Data collection", while authors describe the sources of data used in this study, they do not list the sample size of data downloaded from each source. I recommend that the authors explicitly list the sample sizes for each dataset used in the study, including the number of samples for both bulk RNA-seq and single-cell RNA-seq data from mouse and human sources. A table summarizing sample sizes, their sources and any preprocessing steps would be particularly helpful.**

*Response:* Thanks for the valuable suggestion. To provide a clearer description of "Step 1: Data collection", we provided a table (**Revised S1** **Table**) summarizing the sample sizes, their sources and the preprocessing.

**2. The study integrates data from mouse and human species, including bulk RNA-seq and single-cell RNA-seq data, but it does not provide detailed methods for integrating cross-species data or addressing potential biases between bulk and single-cell data. Specifically, how were homologous genes aligned between mouse and human datasets, and what strategies were used to mitigate platform-specific biases?**

*Response:* Thanks for the valuable suggestion. Homologous genes were aligned using the BioMart database*.* We added homologous gene alignment methods from lines 745 to 748 of the manuscript as follows, “*Homologous genes between mice and humans were aligned using R package biomaRt (v2.46.3) [1]. The Mouse and Human BioMart databases were extracted using the useMart function, followed by alignment of homologous genes using the getLDS function.*”

Second, we used bulk and single-cell RNA-seq in our study. The bulk RNA-seq represented the transcriptome from Fluorescence-activated Cell Sorting (FACS-sorted) cells, while the cell types of scRNA-seq were assigned by the expression of gene markers. To mitigate platform-specific biases, we extracted features separately from bulk RNA-seq and scRNA-seq data. For the bulk RNA-seq data, cell types were sorted based on lineage commitment and assigned consecutive numbers starting from 1 to represent pseudotime. For example, HSC, MPP, GMP, ProNeu, and PreNeu were set as 1, 2, 3, 4, and 5, respectively. For scRNA-seq, the monocle R package (v2.22.0) [2] was used to infer the pseudotime of 2,803 immature and mature neutrophils from the bone marrow dataset [3] with default parameters. Then features including range, slope, and correlation were extracted the same way from bulk RNA-seq and scRNA-seq separately using their respective pseudotime. Features from both platforms were combined and contributed to the model. We revised the manuscript from lines 733 to 748 as follows, “*We extracted 16 features from bulk and scRNA-seq data separately…*”

**3. while the authors mention the use of Monocle for pseudotime calculation, details such as parameter settings and criteria for trajectory inference are missing.**

*Response:* Thanks for pointing out the missing details. We have revised the manuscript from lines 741 to 743 as follows, “*For scRNA-seq, the monocle R package (v2.22.0) [2] was used to infer the pseudotime of 2,803 immature and mature neutrophils from the bone marrow dataset [3] with default parameters.”*

**4. In this part “Then the naïve Bayesian (NB) algorithm used the P set as positive and the U set as negative. The NB classifier was then applied to assign a probabilistic class label to each gene in the U set. The probabilistic labels of the spies were used to decide which genes were most likely to be negative.”, the authors describe using a naïve Bayesian (NB) classifier to predict labels for the unlabeled dataset (U set). However, several critical details are missing. What are the respective sizes of the P set and U set, and how were these datasets partitioned into training and testing sets for training the NB classifier? Additionally, when the authors state, "assign a probabilistic class label to each gene in the U set," does the U set refer to the testing subset or the entire unlabeled dataset? Authors should clarify these points.**

*Response:* Thanks for the comment. We revised PU-learning methods from lines 821 to 834 of the manuscript as follows, "*The positive set (P set) included 293 protein-coding genes with the Gene Ontology (GO) of keyword ‘neutrophil’ and reported in the literature, whereas the negative training set was confirmed by the PU-learning (Positive-Unlabeled Learning) Spy algorithm [4]. In this approach, we randomly selected 10% of the genes (set parameter prob = c(0.9,0.1) in sample function) from the P set to act as "spies" and added them to the Unlabeled set (U set, including 19,581 genes). This created the Us set, which now contained 19,603 genes. The remaining genes from the P set formed the Ps set, which contained 271 genes. To balance the sizes of the sets, we performed oversampling on the Ps set (from 271 to 19,603 genes) using the ovun.sample function from the R package ROSE (v0.0-4)* [5]*, matching the number of genes in the Us set. We then trained a Naive Bayes classifier (G) on the combined Ps+Us training set, Ps as positive and Us as negative, using the classifier G to predict probabilities for both the original U set and the spies. The 10^th^ percentile of the probabilities for spies was used as a threshold, below which genes in the U set were classified as reliable negatives (RN).*"

**5. The authors used Gaussian Mixture Models (GMM) scores to get ‘non-functional’ genes, but the paper does not provide sufficient details about the training process for the GMM. Specifically, what dataset was used to train the GMM, and how were the parameters determined? Additionally, were any validation steps performed to confirm the accuracy of the classification?**

*Response:* Thanks for pointing out the missing details. The GMM is an unsupervised clustering method, that was applied directly to the NeuRGI scores, no external dataset was used. Its parameters are inferred from the data itself through the Expectation-Maximization (EM) algorithm. We added GMM details to the manuscript from lines 868 to 879 as follows, “*A Gaussian Mixture Model (GMM) was applied directly to the NeuRGI scores to characterize the distribution of predicted gene scores. The analysis was performed using the mclust package in R (v6.0.0) [6]. The model was fitted using function Mclust with the parameters G = 3 and modelNames = ‘V’, which models the data as originating from three Gaussian distributions with variable variances for each cluster. The GMM is defined as follows:*

$$p(x)=\sum_{k=1}^{3} \pi_{k}N(x\mid\mu_{k},\Sigma_{k})$$

*Where*$\pi_{k}$ *represents the mixture proportions, and* $N\left( x \mid\mu_{k},\Sigma_{k} \right)$*is the Gaussian distribution with mean* $\mu_{k}$ *and covariance* $\Sigma_{k}$*for each component k. The parameters*$\pi_{k}$*,* $\mu_{k}$*, and* $\Sigma_{k}$ *were estimated from the data using the Expectation-Maximization (EM) algorithm.”*

To validate the classification results, CellOracle [7] and SCENIC [8] were used to assess our positive datasets. The comparison results have been added to the manuscript from lines 177 to 181 as follows:

“*we next compared NeuRGI with CellOracle [7] and SCENIC [8], two state-of-the-art tools for inferring gene regulatory networks (GRN) based on perturbation scores and regulon activity, respectively. Using scRNA-seq data of 2,803 bone marrow neutrophils [3], CellOracle and SCENIC identified 70 and 36 TFs, respectively (****S6 Table****). The majority of these TFs (68.6% for CellOracle and 75.0% for SCENIC) were classified as functional by NeuRGI (****S1E Fig****).”* The methods for CellOracle and SCENIC methods were added to the manuscript from lines 935 to 986 as follows, “*Python package CellOracle (v0.20.0) was used to…Python package pySCENIC (v0.12.1) was used to…*”

Furthermore, we evaluated the predicted functional and non-functional sets regarding the four feature categories, the enrichment of regulatory genes and one gene experimental validation. First, the feature values in the predicted ‘functional’ set were significantly higher than those in the predicted ‘non-functional’ set (**Revised Fig 2C**), consistent with the observation in the model training, validating the accuracy of the classification. Notably, 53.7% of positive genes (2,569) were predicted as enzymes (1,234), membrane proteins (726), TFs (397) or RBPs (212), much higher than the negative gene set (28.1%, **Revised Fig 2E**, proportion test, p = 4.1 x 10^-142^), suggesting the predicted functional set was enriched with regulatory genes, further validating the accuracy of the classification. Finally, we validated *MAP4K4* gene as a regulator of neutrophil differentiation and validated its function through in vivo and in vitro experiments.

Taken together, NeuRGI demonstrated high accuracy in CellOracle and SCENIC in predicting functional regulators particularly those with high pathological relevance. Unlike CellOracle and SCENIC, NeuRGI extends its predictions beyond TFs, capable of identifying a broader range of functional genes. And NeuRGI-predicted functional genes are enriched for regulatory roles, exhibit higher feature scores, and are closely linked to key immune processes. These findings highlight NeuRGI as a comprehensive and robust tool for functional gene inference. The revised results were added to the manuscript from lines 164 to 221.

*Revised S1 (E) Fig. Pie chart illustrating the NeuRGI classification of 70 CellOracle-predicted TFs and 36 SCENIC-predicted TFs.*

*Revised Fig 2 (C). The boxplot illustrates the transformed feature values for 4,786 functional genes and 4,734 non-functional genes in four feature groups. The p value was calculated using the Student’s t-test.*

*Revised Fig 2 (E). Proportion of regulatory (red) and other (blue) genes across three gene sets. Regulatory genes include enzyme (Enz), membrane protein (MP), RNA binding protein (RBP), and transcription factor (TF). The p value was calculated by the proportion test. Numbers within bars represent the gene counts.*

**6. The paper provides a brief description of the OntoVAE training process but lacks critical details necessary for reproducibility. Specifically, the authors should clarify the size of the dataset used for training OntoVAE, the architecture of the encoder and decoder (e.g., the number of layers and the type of networks used), the number of training epochs, and how the loss function was defined and optimized. Providing these details would allow readers to better understand the model's configuration.**

*Response:* Thank you for pointing out the lack of OntoVAE model details. We trained the OntoVAE on the gene expression data of 2040 immune cells, the details of the dataset have been summarized in **Revised S1 Table.** Regarding the OntoVAE model architecture and training details, we have written a **Supplementary Note** (7 pages) to help readers better understand the model and improve its reproducibility. In **Supplementary Note**, we included the architecture of OntoVAE, the loss function, training details, and so on.

**7. While the authors performed feature importance analysis using the Gini coefficient, the paper does not discuss experiments involving the removal of features with low importance scores. Without such analysis, it is unclear whether these less important features contribute noise or negatively impact model performance. The authors should consider conducting feature ablation experiments to assess the effect of removing low-importance features on the model's predictive performance.**

*Response*: Thanks for the suggestion. We conducted feature ablation studies and found that the model performed best after removing 7 bottom-ranked features (Neutrophil to lymphocyte ratio, Sum of neutrophil and eosinophil counts, Neutrophil percentage of leukocytes, Neutrophil percentage of granulocytes, Abnormal neutrophil count, Hom Tau bulk RNA-seq, Cor scRNA-seq, and Hom Spearman.p bulk RNA-seq) (**Revised S1B Fig**). During the ablation process, we observed that removing these features had little effect on AUC and AUC-PR, which remained stable. However, ACC, MCC, F1 score, and the overall mean of the model reached the highest level (**Revised S1C Fig**), suggesting that these features are likely redundant. Therefore, this refined model was established as the NeuRGI model (**Revised S4 Table**). The results have been added to the manuscript from lines 147 to 156 as follows, “*We performed a random forest model*…”.

*Revised S1 (B) Fig. Dot plot showing the feature importance of the baseline model using Gini coefficient with all features included.*

*Revised S1 (C) Fig. Feature ablation studies. The line plots depict changes in the model's evaluation metrics—AUC, AUC-PR, ACC, MCC, F1 Score, and Mean (average of these five metrics)—as features are sequentially removed in reverse order of their importance. The results indicate that the model achieves optimal performance when the last seven features are excluded.*

*Revised S4 Table. Feature ablation studies.*

| Number of deleted features | AUROC | PRROC | ACC | MCC | F1 Score | Mean |
| --- | --- | --- | --- | --- | --- | --- |
| 0 (baseline model-all features) | 0.9776 | 0.9844 | 0.9506 | 0.9043 | 0.9521 | 0.9538 |
| 1 | 0.9779 | 0.9847 | 0.9506 | 0.9039 | 0.9519 | 0.9538 |
| 2 | 0.9753 | 0.9829 | 0.9591 | 0.9195 | 0.9600 | 0.9594 |
| 3 | 0.9762 | 0.9832 | 0.9574 | 0.9161 | 0.9584 | 0.9583 |
| 4 | 0.9763 | 0.9830 | 0.9590 | 0.9193 | 0.9601 | 0.9595 |
| 5 | 0.9759 | 0.9838 | 0.9575 | 0.9168 | 0.9585 | 0.9585 |
| 6 | 0.9777 | 0.9842 | 0.9592 | 0.9208 | 0.9598 | 0.9604 |
| **7** | **0.9773** | **0.9840** | **0.9624** | **0.9268** | **0.9636** | **0.9628** |
| 8 | 0.9782 | 0.9844 | 0.9555 | 0.9130 | 0.9569 | 0.9576 |
| 9 | 0.9773 | 0.9847 | 0.9523 | 0.9068 | 0.9537 | 0.9550 |
| 10 | 0.9775 | 0.9844 | 0.9556 | 0.9138 | 0.9565 | 0.9576 |
| 11 | 0.9768 | 0.9842 | 0.9573 | 0.9164 | 0.9585 | 0.9586 |
| 12 | 0.9773 | 0.9845 | 0.9523 | 0.9070 | 0.9532 | 0.9549 |
| 13 | 0.9756 | 0.9828 | 0.9540 | 0.9102 | 0.9552 | 0.9555 |
| 14 | 0.9743 | 0.9815 | 0.9522 | 0.9064 | 0.9531 | 0.9535 |
| 15 | 0.9751 | 0.9819 | 0.9505 | 0.9037 | 0.9521 | 0.9526 |
| 16 | 0.9785 | 0.9855 | 0.9538 | 0.9105 | 0.9553 | 0.9567 |
| 17 | 0.9745 | 0.9815 | 0.9422 | 0.8869 | 0.9438 | 0.9458 |
| 18 | 0.9743 | 0.9827 | 0.9471 | 0.8960 | 0.9485 | 0.9497 |
| 19 | 0.9703 | 0.9703 | 0.9555 | 0.9128 | 0.9564 | 0.9531 |
| 20 | 0.9673 | 0.9672 | 0.9487 | 0.8989 | 0.9499 | 0.9464 |
| 21 | 0.9643 | 0.9640 | 0.9420 | 0.8854 | 0.9430 | 0.9398 |
| 22 | 0.9528 | 0.9532 | 0.9198 | 0.8427 | 0.9221 | 0.9181 |
| 23 | 0.9519 | 0.9545 | 0.9044 | 0.8119 | 0.9070 | 0.9060 |
| 24 | 0.9257 | 0.9433 | 0.9112 | 0.8293 | 0.9163 | 0.9052 |
| 25 | 0.9239 | 0.9394 | 0.8975 | 0.8046 | 0.9031 | 0.8937 |
| 26 | 0.9236 | 0.9385 | 0.8992 | 0.8080 | 0.9063 | 0.8951 |

**8. The study lacks comparisons with other gene regulatory prediction models, such as CellOracle or SCENIC. Benchmarking against these models would highlight MRGI's unique advantages.**

*Response:* Thanks for the valuable suggestion. We compared NeuRGI with existing gene regulatory predictive models CellOracle [7] and SCENIC [8]. We added the comparison results to the manuscript from lines 177 to 190 as follows:

“*We next compared NeuRGI with CellOracle [7] and SCENIC [8], two state-of-the-art tools for inferring gene regulatory networks (GRN), based on perturbation scores and regulon activity, respectively. Using scRNA-seq data of 2,803 BM neutrophils [3], CellOracle and SCENIC identified 70 and 36 TFs, respectively (****S6 Table****). The majority of these TFs (68.8% for CellOracle and 75.0% for SCENIC) were classified as functional by NeuRGI (****S1E Fig****). Additionally, all of the top 20 TFs ranked by CellOracle’s perturbation score and SCENIC’s regulon activity overlapped with NeuRGI’s predicted functional set (****Fig 2B****), demonstrating accuracy of NeuRGI. Notably, 22 and 9 TFs that were predicted to be important by CellOracle or SCENIC were predicted as “uncertain” by NeuRGI. We further explore the biological distinctions between the ‘uncertain’ and the ‘functional’ TFs called by NeuRGI and one of the other methods. When comparing four feature values across the two groups, we observed that consistently functional TFs showed significantly higher pathological relatedness than uncertain TFs (p < 0.012,* ***S1F Fig****), further showing the accuracy of NeuRGI in predicting functional TFs.*“

The CellOracle and SCENIC methods were added to the manuscript from lines 935 to 986 as follows, “*Python package CellOracle (v0.20.0) was used to…Python package pySCENIC (v0.12.1) was used to…*”

*Revised S1 (E) Fig. Pie chart illustrating the NeuRGI classification of 70 CellOracle-predicted TFs and 36 SCENIC-predicted TFs.*

*Revised Fig 2 (B). The line plot depicts the overlap of functional transcription factors (TFs) predicted by CellOracle and NeuRGI (red line), and by SCENIC and NeuRGI (blue line). The x-axis represents the top N TFs ranked by CellOracle's perturbation score or SCENIC regulon activity. The y-axis shows the percentage of overlap between the predicted functional TFs.*

*Revised S1 (F) Fig. The boxplot illustrates the transformed feature values for 70 CellOracle-predicted TFs (left) and 36 SCENIC-predicted TFs (right) in four feature groups. The p value was calculated using the Student’s t-test.*

**9. The study uses HEK293T cells to validate the function of MAP4K4. However, HEK293T cells are not derived from the hematopoietic or myeloid lineage and exhibit significant differences in biological characteristics compared to myeloid cells or neutrophils. This discrepancy may limit the relevance of the findings to the biological processes under investigation and potentially reduce the credibility of the conclusions. The authors should consider relevant cell lines to provide a more biologically relevant validation model and strengthen the study’s conclusions.**

*Response:* Thanks for the valuable suggestion. In our study, HEK293T cells were used exclusively for the production of viral supernatant but not in the functional validation assays, as described in the section of ‘Viral Transduction’ in Methods. To investigate the role of MAP4K4 in neutrophil differentiation in vitro, we utilized HL-60 cells (**Fig 3A, 3B; Fig 5I, 5J; Fig 6G**). Additionally, to analyze the effects of MAP4K4 on downstream proteins, we conducted protein mass spectrometry (**Fig 6B**), protein phosphorylation mass spectrometry (**Fig 6B to 6E**), and western blot using K562 cells (**Fig 6F**).

**10. When constructing the balanced dataset for training the random forest, the authors downsampled the negative samples to 400, while the positive samples totaled 411. It is unclear why 400 negative samples were chosen instead of 411 to match the exact size of the positive set. This choice may introduce a slight imbalance in the dataset, potentially affecting model training.**

*Response:* Thanks for the suggestion. In the revision, we updated our model and matched positive and negative sample sets with 293 genes. We revised the manuscript from lines 142 to 145 as follows: "*The under-sampling method* [9] *was used to obtain a balanced training data set, consisting of 293 positive and 293 negative genes (****S3 Table****). The GO-BP enrichment analysis of the ‘N’ set indicated that the genes were not related to neutrophil differentiation (****S1A Fig****).*"

**11. The study uses AUC as the sole evaluation metric for model performance. I recommend including additional evaluation metrics, such as PRC (precision-recall curve), F1-score, MCC, and ACC, to provide a more comprehensive assessment of the model’s performance.**

*Response:* Thanks for the suggestion. We have added a variety of evaluation metrics for model performance (**Revised Fig 1B and Revised S4 Table**), and added the results in the manuscript from lines 150 to 156 as follows: “*We found that the model, after removing 7 bottom-ranked features (Neutrophil to lymphocyte ratio, Sum of neutrophil and eosinophil counts, Neutrophil percentage of leukocytes, Neutrophil percentage of granulocytes, Abnormal neutrophil count, Hom Tau bulk RNA-seq, Cor scRNA-seq, and Hom Spearman.p bulk RNA-seq), exhibited the best overall performance (AUC = 0.977, AUC-PR = 0.984, Accuracy = 0.962, MCC = 0.927, F1 Score = 0.964,* ***Fig 1B*** *and* ***S4 Table****).*” The calculation methods of AUC-PR, MCC, ACC, and F1 Score were added in the manuscript from lines 891 to 918 as follows: “*2. Area Under the Precision-Recall Curve (AUC-PR)…*”

*Revised Fig 1 (B). Mean AUC and AUC-PR of the model under 10-fold cross-validation*

**12. The README file currently outlines only the basic framework of this work. It should include detailed instructions on how to use the method, provide a toy example, and offer a clear explanation of the tool's functionality, purpose, and applicable scenarios. Incorporating these elements would greatly enhance its clarity and usability. Additionally, the datasets used in this work should be made available on the GitHub repository to further improve accessibility and comprehensiveness.**

*Response:* Thank you for the constructive comments. We have taken your suggestions seriously and have updated the README file to include detailed instructions, a tutorial, the dataset used in the study, and a clear explanation of the tool's functionality, purpose, and applicable scenarios. The updated README file can be found at the following address: <https://github.com/LuChenLab/NeuRGI.>

**13. There are several grammar mistakes that can easily be corrected with Grammarly etc, for example: “We conducted in vitro studies using HL-60 human leukemia cells, which can be induced to a neutrophil-like (CD11b+) state...”**

*Response:* Thanks for the valuable suggestion. We have modified the sentence in the revised manuscript from lines 277 to 280 as follows:

"*To investigate the role of MAP4K4 in neutrophil differentiation, HL-60 human leukemia cells were used in the in vitro studies to model neutrophil differentiation. HL-60 can be differentiated into neutrophil-like cells characterized by CD11b expression upon stimulation with all-trans-retinoic acid (ATRA).*"

**14. In Fig. 2H and 2I, the capitalization of ligand labels is inconsistent. Please standardize all ligand labels to uppercase (e.g., "TIGAR").**

*Response:* Thanks for your suggestion. The data in **Revised Fig 2I** are from human UCB, hence the genes are capitalized, while the data in **Revised Fig 2J** are from mouse bone marrow, which is why the genes are in lowercase.

**Reviewer #2: The authors developed a machine learning pipeline called myeloid regulatory gene identifier (MRGI), which uses a random forest model to predict potential genes involved in myeloid cell differentiation. Here, a positive unlabeled (PU) learning algorithm was used to train a model where 411 genes belonging to the regulation of myeloid differentiation was used as a positive set. The model predictions not limited to TFs included other genes such as enzymes, membrane proteins, and RBPs. The model predicted three novel genes namely, MAP4K4, FIG4, and TIGAR to be involved in myeloid cell differentiation, of which MAP4K4 was shown to be important for neutrophils. The authors showed that loss of MAP4K4 resulted in neutropenia in mice and MAP4K4 regulated apoptosis in neutrophil differentiation by modulating phosphorylation of STAT5. While MAP4K4 is shown to be a promising target for neutrophil differentiation based on the computational analysis and the experimental validation, given the scope of the journal and its audience, the manuscript could be improved by addressing the follows concerns:**

*Response:* Thank you for your positive comments. Below, we provide point-by-point responses to your comments.

**1. The authors used four broad categories that comprised 41 features in total to train the model. Most of these features are specifically associated with neutrophils, granulocytes, and basophils - cells originating from GMP. In contrast, fewer to none are specifically associated to other myeloid cells such as dendritic cells or macrophages. This is more evident in the physiological characteristics category, the inclusion of leukocyte count may reduce specificity, as leukocytes also include lymphocytes. To improve the model's specificity, I suggest running the model using only neutrophil-associated features or focusing on specific myeloid cells. Furthermore, the authors use genes associated to “regulation of myeloid cell differentiation” comprising of 411 genes as the only positive gene set for the PU-learning algorithm. This can further reduce the specificity. I strongly suggest using features and positive gene sets that are specific to each myeloid cells or better focus only on neutrophils as this would improve sensitivity of the model and align with the later experimental validation.**

*Response:* Thank you for your valuable suggestions. For the dynamic expression features, we calculated various dynamic indicators specific to the neutrophil differentiation process. In terms of physiological and pathological features, we retained only neutrophil-related features. For the selection of the positive gene set, we collected 293 genes from neutrophil-related pathways and from literature[10–13] to enhance the specificity of the model. Using these data and features, we built a neutrophil prediction model. Through feature ablation studies, we selected the model with the best overall performance. At the same time, we reconstructed the best-performing myeloid model using the same approach and compared the two models. We found that the neutrophil model outperformed the myeloid model (**Table R2.1**). Based on your suggestions, we constructed the neutrophil model and renamed our machine-learning pipeline to NeuRGI. (<https://github.com/LuChenLab/NeuRGI>).

*Table R2.1 Comparison of the best myeloid model and the best neutrophil model*

|  | Training set size | Feature  number | AUC | AUC-PR | ACC | MCC | F1 Score |
| --- | --- | --- | --- | --- | --- | --- | --- |
| Myeloid best model | 411(positive)+411(negative) | 27 | 0.947 | 0.965 | 0.889 | 0.783 | 0.894 |
| Neutrophil best model | 293(positive)+293(negative) | 21 | 0.977 | 0.984 | 0.962 | 0.927 | 0.964 |

**2. The authors generate MAP4KA IFN-inducible KO, confirm the delay in differentiation, and analyze the rate of apoptosis in neutrophil subsets. Also, differences in scRNA-seq data, between wt and cKO, appeared to be very nuanced - are the functional properties of MAP4KA KO affected as well? ROS, NET, phagocytosis etc?**

Thanks for the valuable suggestion. According to your suggestion, we examined the neutrophil function-related processes of ROS production and the phagocytic capacity of KO neutrophils. The results indicated that the *Map4k4*-deficiency did not affect the intracellular ROS levels and phagocytosis of neutrophils. These results have been incorporated into the manuscript from lines 348 to 358 as follows:

“*To investigate the whether Map4k4 affects the function of neutrophils, we assessed the reactive oxygen species (ROS) production in neutrophils and the precursors in BM. No significant difference in ROS levels were found among all of these populations in the bone marrow of Map4k4-deficient mice compared to the control mice (****Fig 4E****). Similarly, neutrophils isolated from the spleen and peripheral blood of Map4k4-deficient mice exhibited no significant alterations in ROS production (****S4H Fig****). Furthermore, the phagocytosis of E. coli by neutrophils from both the spleen and peripheral blood also showed no significant differences in the Map4k4-deficient mice (****S4I Fig****). These results suggest that the deletion of Map4k4 primarily affects the differentiation of neutrophils, with no significant impact on their function, such as ROS production and phagocytic activity.*”

*Revised Fig 4 (E).* *ROS generation of neutrophil progenitors in the bone marrow of control (Ctrl) or Map4k4-cKO (cKO) mice. MFI, mean fluorescent intensity;* *ROS, reactive oxygen species; MB, myeloblasts; PM, promyelocytes; MC, myelocytes; MM, metamyelocytes; BC/SC, band cells and segmented neutrophils; (n=6; mean ± SD). Mann-Whitney U test.*

*Revised S4H-I Fig. (H) ROS generation of neutrophils in the spleen (Left) and peripheral blood (Right) of control (Ctrl) or Map4k4-cKO (cKO) mice; MFI, mean fluorescent intensity; ROS, reactive oxygen species; (n=6; mean ± SD). (I) The percentage of phagocytic neutrophils in the spleen (Left) and peripheral blood (Right) of control (Ctrl) or Map4k4-cKO (cKO) mice; (n=6; mean ± SD). Mann-Whitney U test.*

**Minor comments:**

**3. Line 87-91. All features or any of the four features? Elaborate.**

*Response:* Thanks for your suggestion. We reasoned that a functional or driver gene should have all four features. We revised the manuscript from lines 109 to 114 as follows:

“*To identify genes important in neutrophil differentiation, we developed a supervised learning algorithm NeuRGI (Neutrophil Regulatory Gene Identifier) (****Fig 1A****). We reasoned that a functional or driver gene should* ***have all of the following four features****: (1) differential or dynamic expressed during neutrophil differentiation. (2) associated with various physiological characteristics of neutrophils. (3) associated with related diseases. (4) conserved in multiple species.*"

**4. Line 93-97. How are the datasets between mouse and human datasets integrated or compared?**

*Response:* Thanks for your suggestion. The datasets from mice and humans were not directly integrated or compared. Instead, they were utilized separately to calculate expression dynamics features. Then homologous genes between mice and humans were aligned using the BioMart database [1]*.* We revised the manuscript from lines 116 to 121 as follows: *“We collected extensive datasets for feature extraction (****S1 Table****). The expression dynamics were calculated using three RNA-sequencing datasets* ***independently*** *including bulk RNA-seq data of 8 FACS-sorted continuous hematopoietic populations in mouse bone marrow* [14] *and human umbilical cord blood* [15]*, and scRNA-seq data of sorted CD11b+Ly6G+ mouse neutrophils [3].* ***Homologous genes between mouse and human were aligned using BioMart database*** *[1].”*

We also added homologous genes alignment details to the manuscript from lines 745 to 748 as follows, “*Homologous genes between mice and humans were aligned using R package biomaRt (v2.46.3) [1]. The Mouse and Human BioMart databases were extracted using the useMart function, followed by alignment of homologous genes using the getLDS function.”*

**5. Line 99-100. Elaborate further, are the dynamic and differential analysis done on all myeloid cells or specifically neutrophils and their progenitors? Please include details.**

*Response:* Thanks for the valuable suggestion. In the initial manuscript, a dynamic and differential analysis was conducted on all myeloid cells. However, upon careful consideration of your valuable suggestion, we have recalculated the expression dynamics exclusively for neutrophils and their progenitors. Utilizing these refined features, we have retrained our model to enhance its predictive accuracy. Furthermore, we have updated the description in the manuscript to reflect these methodological improvements. We revised the manuscript from lines 121 to 124 as follows:

"*We calculated features such as gene expression range, cell specificity index Tau, and gene expression change rate extracted from the pseudotime trajectory inferred from scRNA-seq, to measure the differential and dynamic gene expression fluctuations throughout* ***neutrophil differentiation****.*"

**6. Line 113/Table S2. Would be useful to include the type of gene as a column in table S2, does the proportion of the type in the positive/negative set influence the type of gene as seen in Figure 2E. Essentially, is the MRGI score of enzymes significantly higher than other protein types because there are more enzymes in the training data?**

*Response:* Thank you for your valuable suggestion. We have included the type of gene as a column in the **Revised S3 Table**. Below is a portion of the **Revised S3 Table**.

After training the model using only the neutrophil-related positive set, we checked the distribution of protein types in the training data. The number of proteins of each type in both the positive and negative training sets is the same, as follows: Other (131), TF (53), MP (50), Enz (46), and RBP (13). Although the number of enzymes (Enz) is fewer than that of most other protein types, except for RBPs, **Revised Fig 2F** still shows that the NeuRGI score of enzymes is significantly higher than that of the other protein types, suggesting that a higher protein count does not necessarily correlate with a higher NeuRGI score.

*Revised Fig 2 (F). Boxplot of NeuRGI scores, color-coded by gene type: Enzyme (Enz), membrane proteins (MP), RNA binding proteins (RBP), Transcription factors (TF), and others. The p value was calculated using the Student’s t-test.*

**7. Line 119. Kindly specify the type of GO analysis used here.**

*Response:* Thanks for your suggestions. Gene Ontology Biological Processes (GO-BP) term enrichment analysis has been clarified and updated in the manuscript.

**8. Line139. How are these peak ranges defined? Unclear.**

*Response:* Thanks for your suggestion. These peak ranges are defined by R package quantmod (v0.4.26) [16]. We revised the manuscript from lines 167 to 169 as follows, “*We observed that the distribution of the NeuRGI score exhibited a bimodal distribution, with two peaks around 0.02 and 0.97 identified using R package quantmod (v0.4.26) [16], respectively (****Fig 2A****)*”. We also added quantmod details to the manuscript from lines 868 to 869 as follows, “*The ranges of the two peaks in the NeuRGI score density were determined using the findPeaks function from the R package quantmod (v0.4.26) [16].* "

**9. Line 154. Fig2B. Would be interesting to see this figure for all features in Table S1.**

*Response:* Thanks for your valuable suggestions, we have examined the transformed feature values of all characteristics for both the predicted functional and predicted non-functional categories by NeuRGI. We have found that, except the 'Mus Spearman.cor of bulk RNA-seq' which shows no significant difference, all other features exhibit significant differences (**Fig R2.1**).

*Fig R2.1 The boxplot illustrates the transformed feature values for 4,786 functional genes and 4,734 non-functional genes in all the features. The p value was calculated using the Student’s t-test.*

**10. Line 161. Kindly specify the type of GO analysis used here.**

*Response:* Thanks for your suggestions. Gene Ontology Biological Processes (GO-BP) term enrichment analysis has been clarified and updated in the manuscript.

**11. Line 171-177. Explanation insufficient, to improve readability, kindly elaborate further on OntoVAE, the datasets used, and the accuracy over the predictions.**

*Response*: Thanks for pointing out the lack of clarity in the manuscript. We have written a **Supplementary Note** to help readers better understand OntoVAE. The datasets used in OntoVAE were gene expression data of 2,040 immune cells, which had been summarized in the **Revised S1 Table**. For the accuracy of prediction, we performed *in silico* knockout of *ELANE* [17] and *SYK* [18] in neutrophils and B cells, two genes have been reported to play important roles in neutrophils and B cells, respectively. We found that pathways related to neutrophils and B cells terms were significantly affected in consistent with previous studies[19–22] (**Revised S2A Fig**), suggesting that using the data in the **Revised S1 Table**, together with the OntoVAE, can accurately mimic the *in silico* knockouts. We have revised the manuscript from lines 225 to 240 as follows, “*We performed in silico knockout in neutrophils for the 2,569 genes using Ontology-guided Variational Autoencoder (OntoVAE)…*”

*Revised S2 (A) Fig. In silico knockout of ELANE and SYK in neutrophils and B cells, respectively. Bar plots display the affected neutrophils and B cells related terms ranked by significance. The dotted line represents the significance threshold.*

**12. Fig3A – Scale is missing.**

*Response:* Thanks for the valuable suggestion. We revised the figure as follows:

*Revised Fig 3 (A). Scheme of all-trans-retinoic acid (ATRA)-induced neutrophil differentiation of the hematopoietic HL-60 cell models (left); flow cytometry analysis of the neutrophil marker CD11b has validated that ATAR can induce differentiation of HL-60 cells into neutrophils. This is demonstrated by histogram plots showing variations in mean fluorescence intensity (MFI) across different conditions: unstained cells, cells untreated with ATAR, cells treated with ATAR for 72 hours, and cells treated with ATAR for 96 hours (right).*

**13. Fig 3B – At what time point was the inhibitor given?**

*Response:* We already added the information to the figure legend of **Fig 3B** in the manuscript from lines 606 to 609 as follows:

"*HL-60 cells were treated with 0-20 µM MAP4K4 inhibitor (PF-06260933, PF) for 48 hours, followed by a subsequent 96-hour treatment with both PF and all-trans-retinoic acid (ATRA).*"

**14. Line 256. Elaborate on the batch correction method used.**

*Response:* Thanks for the valuable suggestion. Anchor genes between Ctrl and *Map4k4*-cKO samples were first identified by the function FindIntegrationAnchors (normalization.method = ‘LogNormalize’ and reduction = ‘rpca’) of R package Seurat (v4.3.0) to remove the batch effect. Then these anchors were used to integrate the two samples with the function IntegrateData. We also revised the manuscript from lines 1184 to 1192 as follows,

"*Anchor genes between Ctrl and Map4k4-cKO samples were first identified by the function FindIntegrationAnchors (normalization.method = ‘LogNormalize’ and reduction = ‘rpca’) of R package Seurat (v4.3.0). These anchor genes represent genes whose expression profiles are shared between the samples, allowing us to align them and remove systematic differences caused by batch effects. With these anchors, we integrated the Ctrl and Map4k4-cKO samples using the IntegrateData function, which merges the datasets while preserving biological variation and correcting for unwanted batch effects.* "

**15. Fig6D. Kindly detail other pathways that are differential between KO and WT.**

*Response:* Thanks for your suggestion. We performed Gene Ontology Biological Processes (GO-BP) enrichment analysis of differential phosphorylation level proteins between Ctrl and *MAP4K4* KO samples. We revised the manuscript from lines 429 to 435 as follows:

“*Then, we performed GO-BP analysis of differential phosphorylation level proteins between Ctrl and MAP4K4 KO samples. Notably, proteins with downregulated phosphorylation levels were specifically enriched with ‘Positive regulation of apoptotic process’ and ‘Positive regulation of myeloid leukocyte cytokine production involved in immune response’, while those up-regulated proteins were uniquely enriched with ‘Mitochondrial ATP synthesis coupled electron transport’ and 'Nucleotide phosphorylation' (****S6E Fig****).”*

***Revised S6 (E) Fig.*** *GO-BP enrichment analysis of differential phosphorylation level proteins between Ctrl and MAP4K4 KO samples. Selected GO terms with Benjamini-Hochberg-corrected p values < 0.05 (one-sided Fisher’s exact test) are shown. The dot size represented the number of genes. The color scale represented the adjusted p value.*

References

1.	Durinck S, Spellman PT, Birney E, Huber W. Mapping identifiers for the integration of genomic datasets with the R/Bioconductor package biomaRt. Nat Protoc. 2009;4: 1184–1191. doi:10.1038/nprot.2009.97

2.	Qiu X, Mao Q, Tang Y, Wang L, Chawla R, Pliner HA, et al. Reversed graph embedding resolves complex single-cell trajectories. Nat Methods. 2017;14: 979–982. doi:10.1038/nmeth.4402

3.	Ng MSF, Kwok I, Tan L, Shi C, Cerezo-Wallis D, Tan Y, et al. Deterministic reprogramming of neutrophils within tumors. Science. 2024;383: eadf6493. doi:10.1126/science.adf6493

4.	Bhardwaj N, Gerstein M, Lu H. Genome-wide sequence-based prediction of peripheral proteins using a novel semi-supervised learning technique. BMC Bioinformatics. 2010;11: 1–8. doi:10.1186/1471-2105-11-S1-S6

5.	Menardi G, Torelli N. Training and assessing classification rules with imbalanced data. Data Min Knowl Discov. 2014;28: 92–122. doi:10.1007/s10618-012-0295-5

6.	Scrucca L, Fop M, Murphy TB, Raftery AE. mclust 5: Clustering, Classification and Density Estimation Using Gaussian Finite Mixture Models. R J. 2016;8: 289–317.

7.	Kamimoto K, Stringa B, Hoffmann CM, Jindal K, Solnica-Krezel L, Morris SA. Dissecting cell identity via network inference and in silico gene perturbation. Nature. 2023;614: 742–751. doi:10.1038/s41586-022-05688-9

8.	Aibar S, González-Blas CB, Moerman T, Huynh-Thu VA, Imrichova H, Hulselmans G, et al. SCENIC: single-cell regulatory network inference and clustering. Nat Methods. 2017;14: 1083–1086. doi:10.1038/nmeth.4463

9.	Xu Q, Wang Z, Jiang C, Liu Y. Deep learning on mixed frequency data. J Forecast. 2023;42: 2099–2120. doi:https://doi.org/10.1002/for.3003

10.	Khoyratty TE, Ai Z, Ballesteros I, Eames HL, Mathie S, Martín-Salamanca S, et al. Distinct transcription factor networks control neutrophil-driven inflammation. Nat Immunol. 2021;22: 1093–1106. doi:10.1038/s41590-021-00968-4

11.	Grieshaber-Bouyer R, Radtke FA, Cunin P, Stifano G, Levescot A, Vijaykumar B, et al. The neutrotime transcriptional signature defines a single continuum of neutrophils across biological compartments. Nat Commun. 2021;12: 2856. doi:10.1038/s41467-021-22973-9

12.	Hackert NS, Radtke FA, Exner T, Lorenz H-M, Müller-Tidow C, Nigrovic PA, et al. Human and mouse neutrophils share core transcriptional programs in both homeostatic and inflamed contexts. Nat Commun. 2023;14: 8133. doi:10.1038/s41467-023-43573-9

13.	Ai Z, Udalova IA. Transcriptional regulation of neutrophil differentiation and function during inflammation. J Leukoc Biol. 2020;107: 419–430. doi:10.1002/JLB.1RU1219-504RR

14.	Wang F, He J, Liu S, Gao A, Yang L, Sun G, et al. A comprehensive RNA editome reveals that edited Azin1 partners with DDX1 to enable hematopoietic stem cell differentiation. Blood. 2021;138: 1939–1952. doi:10.1182/blood.2021011314

15.	Chen L, Kostadima M, Martens JHA, Canu G, Garcia SP, Turro E, et al. Transcriptional diversity during lineage commitment of human blood progenitors. Science. 2014;345: 1251033. doi:10.1126/science.1251033

16.	Ryan JA UJ. quantmod: Quantitative Financial Modelling Framework. R Packag version 04261. 2025.

17.	Rao S, Yao Y, Soares de Brito J, Yao Q, Shen AH, Watkinson RE, et al. Dissecting <em>ELANE</em> neutropenia pathogenicity by human HSC gene editing. Cell Stem Cell. 2021;28: 833-845.e5. doi:10.1016/j.stem.2020.12.015

18.	Zhang S, Wang L, Lu Y, Guo C, Zhang T, Zhang L. Targeting spleen tyrosine kinase (SYK): structure, mechanisms and drug discovery. Drug Discov Today. 2025;30: 104257. doi:https://doi.org/10.1016/j.drudis.2024.104257

19.	Weinrauch Y, Drujan D, Shapiro SD, Weiss J, Zychlinsky A. Neutrophil elastase targets virulence factors of enterobacteria. Nature. 2002;417: 91–94. doi:10.1038/417091a

20.	Nanua S, Murakami M, Xia J, Grenda DS, Woloszynek J, Strand M, et al. Activation of the unfolded protein response is associated with impaired granulopoiesis in transgenic mice expressing mutant Elane. Blood. 2011;117: 3539–3547. doi:10.1182/blood-2010-10-311704

21.	Ackermann JA, Nys J, Schweighoffer E, McCleary S, Smithers N, Tybulewicz VLJ. Syk tyrosine kinase is critical for B cell antibody responses and memory B cell survival. J Immunol. 2015;194: 4650–4656. doi:10.4049/jimmunol.1500461

22.	Chen L, Monti S, Juszczynski P, Daley J, Chen W, Witzig TE, et al. SYK-dependent tonic B-cell receptor signaling is a rational treatment target in diffuse large B-cell lymphoma. Blood. 2008;111: 2230–2237. doi:10.1182/blood-2007-07-100115
