# Supplementary material for: Machine learning-based prediction reveals kinase MAP4K4 regulates neutrophil differentiation through phosphorylating apoptosis-related proteins
Source: PLoS Comput Biol. 2025 Mar 17;21(3):e1012877. doi: 10.1371/journal.pcbi.1012877 (PMC11957395; doi:10.1371/journal.pcbi.1012877)
Supplement: Attachment — Submitted filename: Response_to_reviewers_auresp_2.docx [file pcbi.1012877.s025.docx]

Lu Chen, Ph.D. Professor

Sichuan University, Chengdu, China 610041

Email: [luchen@scu.edu.cn](http://luchen@scu.edu.cn) Tel: (+86)18180609879

Feb. 11, 2025

**MS Submission ID:** PCOMPBIOL-D-24-01958
**MS title:** Machine learning-based prediction reveals kinase MAP4K4 regulates neutrophil differentiation through phosphorylating apoptosis-related proteins

Dear Dr. Murphy and Dr. Rangamani,

We would like to extend our heartfelt thanks to your invaluable assistance and express our gratitude to the reviewers for their positive comments and constructive suggestions. To address the concerns of you and the reviewers, we updated the GitHub repository <https://github.com/LuChenLab/NeuRGI>, revised the supplementary table and discussion section of the manuscript.

We have uploaded a point-by-point response to the reviewers' comments, along with the revised manuscript (both clean and track-change versions). Additionally, we have formatted the manuscript according to the *PLOS Computational Biology* article style, as recommended.

Thank you again for your kind consideration, detailed instructions and continued support throughout the review process. We hope that the revision will satisfactorily address the comments and concerns of you and the reviewers.

Yours Sincerely,

Lu Chen (on behalf of all co-authors)

Professor, Sichuan University

**Point-by-point response**

**Editor**

**Both reviewers felt that you had addressed their major concerns but that some minor issues remain. Please address each of these in your resubmission.**

*Response:* Thanks for the positive comments on our work. We respond to each point raised by the reviewers.

**Reviewer #1**

**The authors have done a thorough job addressing the previous comments. However, there is still one remaining concern. For full reproducibility, all the datasets used in this study should be made available in the GitHub repository, rather than providing only example datasets. Additionally, the notebook OntoVAE.ipynb cannot currently be executed because certain files (e.g., data/GO_symbol_ontobj.pickle) are missing from the repository. Please ensure that every file required by OntoVAE.ipynb is included in the repository. Finally, it would be highly beneficial to provide the trained model checkpoints used in the paper so that the results can be fully reproduced.**

*Response:* Thanks for the positive comments. All datasets, as well as the usage details for the myeloid or neutrophil-specific model, are available in the GitHub repository. Due to GitHub's file size limitations, four essential files required by OntoVAE.ipynb, along with the trained model checkpoints used in the paper are uploaded to the figshare repository <https://figshare.com/articles/dataset/NeuRGI-OntoVAE/28374878>. All relevant updates have been reflected in the GitHub repository <https://github.com/LuChenLab/NeuRGI>. The updated sections of the repository are outlined below (Fig R1.1).

Fig R1.1 The updated sections of the GitHub repository.

**Reviewer #2**

**We thank the authors for making the necessary changes in the first round of revision, the manuscript has improved in clarity and reads well now. Following are some minor comments to improve further.**

*Response:* Thanks again for your comments on our work. For the new comments we have addressed as follows.

**1. Please use consistent capitalization for gene names. For eg., Either MAP4K4 or Map4k4, currently there seems to be a mix of both.**

*Response:* The reason for the inconsistency in the manuscript is that we analyzed the gene in both humans and mice.

According to the nomenclature guidelines for genes and proteins, Human gene symbols are italicized and presented in all capital letters (*MAP4K4*); mouse gene symbols are italicized with only the first letter capitalized (*Map4k4*); and the protein symbols for both humans and mice are identical to their respective gene symbols but are without italicized and are written in all capital letters (MAP4K4). Therefore, in the machine learning section, we used *MAP4K4*. For experiments using human cell lines, we also used *MAP4K4*, such as "*MAP4K4*-KO K562 cells" et al. For the mouse experiments, we used *Map4k4*, such as "*Map4k4*-cko mice" and "*Map4k4*^f/f^ mice”. For protein experiments, we used MAP4K4, such as “Western blot for MAP4K4” et al.

We have gone through the text and ensured the correct use in all sections.

**2. Line 136: Please mention in text the total number of pathways that went into the positive gene set. If not already done, in supplementary please tabulate the neutrophil associated pathways and their associated genes. Also include genes taken from literature (with reference). This would be helpful for addition of newly identified neutrophil-associated genes to the positive gene set in the future.**

*Response:* We counted the total number of pathways that contribute to the positive gene set and revised the text from lines 134 to 137 as follows:

“*To train the model, we encompassed a positive gene set (P set), containing 293 protein-coding genes from 19 Gene Ontology Biological Processes (GO-BP) pathways with the keyword ‘neutrophil’ or genes reported having neutrophil-related functions in the literature* [1–5] *(S3 Table).”*

Additionally, we included a supplementary table (**Revised S3** **Table**) summarising the gene types and sources of the known positive and PU-learning negative training gene sets of NeuRGI using PMID for literature and GO term ID for pathways (a fraction of the S3 Table shown below).

**3. Line 225 & 562 is unclear: Why was the *in silico* KO performed on 2569 genes? Isn’t it supposed be 4786, as reported in line 173-175? I hope this is a typo. The 2569 genes comprise only of enzymes (line 210). However, your resulting 12 genes (lines 257) comprise of other types of genes. Please explain/correct.**

Response: Thanks for the comments on our work. The *in silico* KO was performed on 2,569 genes. In total, we identified 4,786 genes likely to be functional in neutrophil differentiation, as reported in lines 173–175. Among these, 53.7% (2,569 genes) were predicted to encode regulatory proteins, including enzymes (1,234), membrane proteins (726), transcription factors (TFs, 397), and RNA-binding proteins (RBPs, 212), while the remaining 46.3% were classified as other categories (Fig. 2E, revised lines 210–214). We then performed *in silico* knockout on these 2,569 regulatory genes using the Ontology-guided Variational Autoencoder (OntoVAE), as reported in line 227. Therefore, it is consistent that the resulting 12 genes (line 257) include enzymes, membrane proteins, and RBPs. To be clear, we revised the text from lines 210 to 214 as follows:

“*Notably, among the 4,786 genes identified as likely functional in neutrophil differentiation, 53.7% (2,569) genes were predicted to encode regulatory proteins, including enzymes (1,234), membrane proteins (726), transcription factors (TFs, 397), and RNA-binding proteins (RBPs, 212). This proportion is significantly higher than that in the negative gene set (28.1%, Fig 2E, proportion test, p = 4.1 x 10^-142^), suggesting the predicted functional set was enriched with regulatory genes*”.

**4. The third limitation in discussion (line 513-515) seems contradictory to the final statement (line 518-520). The authors can briefly propose methods to extend the approach to other lymphoid and erythroid differentiation states. Such as cell-specific features, positive gene-sets, etc.**

*Response:* Thanks for the suggestions. We revised the discussion from lines 518 to 524 as follows:

“*Third, the specificity of the data used in this study makes NeuRGI tailored for the neutrophil in myeloid linage but not for other hematopoietic lineages, such as lymphoid and erythroid lineages. However, this limitation can be addressed by incorporating lineage-specific positive gene sets, along with features, such as single-cell epigenomics and proteomics* [6,7]*, that capture the unique characteristics of each lineage. Thus, the paradigm of our study can be extended to other lineages or other tissues.*”

**5. The authors mention TF’s such as PU.1, CEBPA, and GFI-1 in their introduction. It would be interesting to see their NeuRGI scores. Essentially, do the well know TF’s important for neutrophil maturation/function also have high scores? If so, this can serve as an independent validation of the method. Worth including in the discussion.**

*Response:* Thanks for the suggestion. The high NeuRGI scores for well-known neutrophil-related TFs, such as *PU.1* (0.9991), *GFI-1* (0.9983), and *CEBPA* (0.9976), further validate the accuracy of the method. We revised the discussion from lines 489 to 492 as follows:

“*Third, our model demonstrates outstanding performance, achieving an AUC of 0.977. Specifically, it not only validates well-known TFs with high scores, such as PU.1, GFI-1 and CEBPA (with NeuRGI scores of 0.9991, 0.9983, and 0.9976, respectively), but also predicts the novel gene MAP4K4, which was further experimentally validated.*”

**References**

1.	Khoyratty TE, Ai Z, Ballesteros I, Eames HL, Mathie S, Martín-Salamanca S, et al. Distinct transcription factor networks control neutrophil-driven inflammation. Nat Immunol. 2021;22: 1093–1106. doi:10.1038/s41590-021-00968-4

2.	Grieshaber-Bouyer R, Radtke FA, Cunin P, Stifano G, Levescot A, Vijaykumar B, et al. The neutrotime transcriptional signature defines a single continuum of neutrophils across biological compartments. Nat Commun. 2021;12: 2856. doi:10.1038/s41467-021-22973-9

3.	Hackert NS, Radtke FA, Exner T, Lorenz H-M, Müller-Tidow C, Nigrovic PA, et al. Human and mouse neutrophils share core transcriptional programs in both homeostatic and inflamed contexts. Nat Commun. 2023;14: 8133. doi:10.1038/s41467-023-43573-9

4.	Ai Z, Udalova IA. Transcriptional regulation of neutrophil differentiation and function during inflammation. J Leukoc Biol. 2020;107: 419–430. doi:10.1002/JLB.1RU1219-504RR

5.	Ai Z. Revealing key regulators of neutrophil function during inflammation by re-analysing single-cell RNA-seq. PLoS One. 2022;17: e0276460. doi:10.1371/journal.pone.0276460

6.	Schwartzman O, Tanay A. Single-cell epigenomics: techniques and emerging applications. Nat Rev Genet. 2015;16: 716–726. doi:10.1038/nrg3980

7.	Single-cell proteomics: challenges and prospects. Nat Methods. 2023;20: 317–318. doi:10.1038/s41592-023-01828-9
